# Urban wastewater and sewage sludge as environmental sources of antimicrobial resistance, potential pathogens, and selective agents: insights from three European wastewater treatment plants

**DOI:** 10.3389/fcimb.2026.1916091

**Published:** 2026-09-10

**Authors:** Anita Solem, Gunhild Hageskal, Anita N. Jakobsen, Sigrid Hakvåg, Ioannis S. Boziaris, Andy M. Booth, Foteini F. Parlapani, Dimitrios A. Anagnostopoulos, Tone Haugen, Terkel Hansen, Andreas H. Tømmerdal, Tonje M. B. Heggeset, Evangelia A. Karamani, Stavroula Letsiou, Sunniva Hoel

**Affiliations:** 1 Department of Biotechnology and Food Science, Norwegian University of Science and Technology, Trondheim, Norway; 2 Department of Biotechnology and Nanomedicine, SINTEF Industry, Trondheim, Norway; 3 Department of Climate and Environment, SINTEF Ocean, Trondheim, Norway; 4 Department of Ichthyology and Aquatic Environment, University of Thessaly, Volos, Greece

**Keywords:** antimicrobial resistance (AMR), marine environment, One Health, sewage sludge (biosolids), wastewater

## Abstract

Antimicrobial resistance (AMR) is a growing global human health threat, and it is increasingly recognized as a One Health issue, complicated by the complex interactions between humans, animals, and the environment. Wastewater treatment plants (WWTPs) link human activities with the environment and may act as a reservoir of AMR, facilitating environmental dissemination. This study investigated bacterial communities, antibiotic resistance genes (ARG), and antibiotic susceptibility in wastewater and digested sludge at three WWTPs in Norway and Greece, representing regions with low and high antibiotic consumption and clinical AMR prevalence. Antibiotic residues and potentially toxic metals were also quantified. Of 21 antibiotics analyzed, 13 were detected in at least one sample, with ciprofloxacin, cefotaxime, and meropenem occasionally exceeding predicted no-effect concentrations. Removal efficiencies varied, with limited removal at the Norwegian WWTPs compared with the Greek facility. Potentially toxic metals were detected at all sites, with exceeded minimum co-selective concentrations for Cu, Ni, Pb, and Zn. Bacterial community composition was influenced by location, sample type, and sampling time point (p<0.05), and was associated with antibiotic concentrations. Removal of fecal and environmental indicator bacteria was 3.1 ± 1.4 log CFU/100 mL at the Greek WWTP but negligible at the Norwegian WWTPs. Most ARGs (79%) were detected across all WWTPs, and clinically important resistance genes were also prevalent in outlet and digested sludge samples, suggesting considerable environmental spread. Antibiotic susceptibility testing revealed reduced susceptibility to all tested antibiotics, including 3^rd^ generation cephalosporins, meropenem, and last-resort antibiotics, although enumeration and susceptibility testing of environmental indicator bacteria was limited by methodological constraints. These findings demonstrate that WWTPs can contribute to the environmental dissemination of ARB and ARG in both high and low antibiotic consumption and clinical AMR regions, highlighting the need for effective wastewater treatment, improved environmental surveillance and integrated One Health approaches linking human and environmental AMR compartments.

## 1 Introduction

With the development and spread of antimicrobial resistance (AMR), the efficiency of antimicrobial treatments is declining, and human health is threatened. AMR was associated with an estimated 4.95 million deaths worldwide in 2019 (Murray et al., 2022) and is projected to be associated with 8.22 million deaths annually by 2050 if no preventive measures are implemented (Naghavi et al., 2024). While antibiotic use and misuse are known drivers of AMR (Bell et al., 2014), its emergence and spread are also shaped by the complex interplay between humans, animals and the environment (Bengtsson-Palme et al., 2018). Consequently, AMR should be addressed within a One Health-framework that recognizes this interconnectedness (Robinson et al., 2016).

Standing at the interface between humans and the environment, urban wastewater may play a central role in the spread of AMR by facilitating interaction between diverse human-associated and environmental bacteria (Guo et al., 2019). Continuous inputs of antibiotics and antibiotic resistant bacteria (ARB; Voigt et al., 2020), together with high bacterial density may provide favorable conditions for the spread of antibiotic resistance genes (ARGs) through horizontal gene transfer (Rizzo et al., 2013). Recent evidence suggests that the initial ARG mobilization occurs through the co-occurrence of species carrying ARGs and mobilizing elements in wastewater environments, rather than in the human gut (Berglund et al., 2023). Antibiotic residues and potentially toxic metals (PTMs) may further promote AMR by exerting selective pressure on the bacterial communities (Seiler and Berendonk, 2012; Stanton et al., 2020), even at levels well below minimal inhibitory concentrations (MICs; Gullberg et al., 2011). Predicted no effect concentrations (PNECs) for resistance selection that may guide risk assessment has therefore been estimated (Bengtsson-Palme and Larsson, 2016). Co-occurrence of two antibiotics, or one antibiotic and one heavy-metal, has further been shown to lower individual minimal selective concentrations (MSCs; Gullberg et al., 2014), although the significance of these interactions in complex wastewater environments remain largely unknown.

Despite several treatment steps, ARB, ARGs, multi-drug resistant bacteria, and WHO bacterial priority pathogens are frequently detected in wastewater effluents (Oravcova et al., 2017; Blaak et al., 2021; Uluseker et al., 2021; Rocha et al., 2022; Grevskott et al., 2024; WHO, 2024), highlighting wastewater treatment plants (WWTPs) as potential contributors to AMR dissemination. Wastewater discharges can increase ARG abundance and diversity, as well as ARB prevalence in receiving waters (Mukherjee et al., 2021; Read et al., 2024), and also alter the bacterial community composition (Wang et al., 2022; Dai et al., 2023). As microbial community structure is tightly linked to the resistome in soil and urban wastewater communities (Forsberg et al., 2014; Ju et al., 2019), it may play a critical role in modulating the persistence and spread of AMR in WWTPs and downstream environments.

During wastewater treatment, biomass accumulate in the sludge fraction, resulting in potentially higher concentrations of ARB than in treated wastewater. Although the agricultural use of urban wastewater sludge is strictly regulated in the EU with regards to the concentration of fecal indicator bacteria, PTMs and organic pollutants (Directive 86/278/EEC, 1986), ARB and ARGs are not monitored or regulated. Furthermore, our understanding of their occurrence and persistence in sludge and sludge-amended soils remain limited. Thus, the potential for AMR spreading through sludge reuse cannot be excluded.

Monitoring of ARB alongside ARGs in wastewater may provide an effective measure of the current AMR situation (Punch et al., 2025). Many studies of ARB in wastewater focus on fecal indicator bacteria, despite evidence that they represent only a small fraction of the wastewater microbiome and do not typically persist in downstream aquatic environments (Newton et al., 2015; Mattioli et al., 2017). This suggests that reliance on fecal indicators alone may underestimate ARB spread. Bacteria such as *Aeromonas* and *Pseudomonas*, which occur across human, wastewater, and receiving environments, have therefore been proposed as more representative indicators of AMR spread across aquatic environments (Milligan et al., 2023).

This study investigates AMR in three WWTPs in Norway and Greece, representing regions with contrasting antibiotic consumption and clinical resistance rates (ECDC, 2024a, b). Antibiotic residues, PTMs, bacterial communities, including ARB and potential pathogens, and resistomes were assessed and characterized in the influent, effluent, and digested sludge from the three WWTPs. Through combined screening of > 2,700 unique ARGs using hybrid capture-based next generation sequencing and high-throughput robotic phenotypical susceptibility testing of bacterial isolates, the study investigates relationships between contaminants, microbial communities, and AMR. The findings provide new insights into the role of WWTPs in the spread of AMR and pathogens within a One Health perspective and may support monitoring decisions and guide wastewater management strategies.

## 2 Methods

### 2.1 Wastewater treatment plants

**Figure 1:**
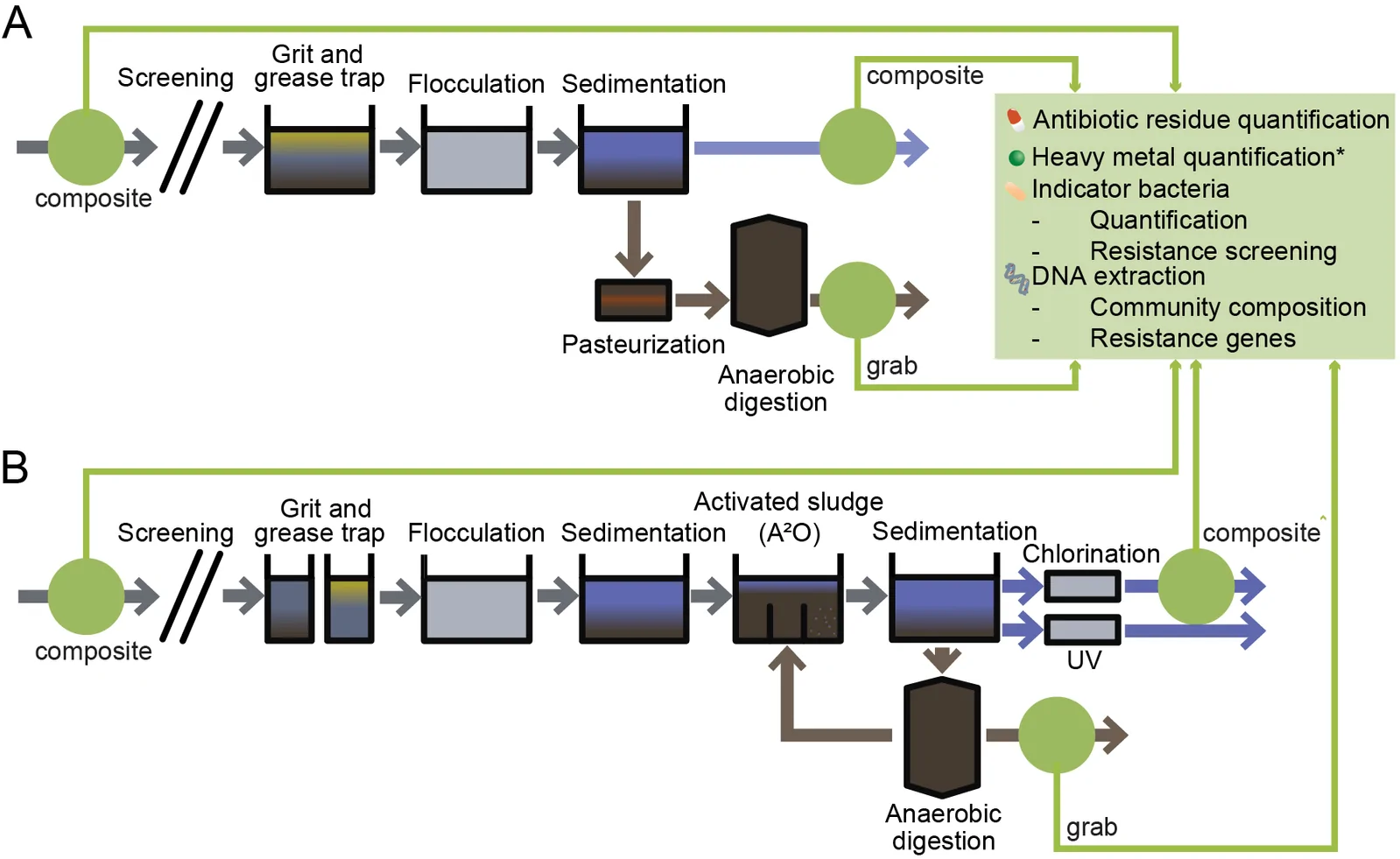
Simplified process flow diagrams for the WWTPs **(A)** NO1 and NO2, and **(B)** GR1. Gray, blue, and brown arrows represent untreated wastewater, treated wastewater, and sludge, respectively. Green circles represent sampling points, and the green box shows analyzed parameters.

Three WWTPs were investigated. NO1 and NO2 are located on the coast of central Norway, while GR1 is located on the coast in the mid-north region of Greece. All three WWTPs serve urban catchment areas and receive wastewater from households, industry, and urban runoff. In addition, NO1 and GR1 receive wastewater from hospitals. Simplified process flow diagrams for the three WWTPs are shown in **Figure 1**.

NO1 and NO2 are chemically enhanced primary treatment facilities with capacities of 170,000 and 120,000 person equivalents (PE), respectively, and annual treatment volumes of approximately 23 and 12 Mm^3^. The treatment reduces the biochemical oxygen demand over 5 days (BOD5) by more than 50% and suspended solids (SS) by more than 70% (**Supplementary Table 1**). After removal of coarse particles, grease, and grit, flocculating chemicals are added, followed by sedimentation. Treated wastewater is discharged into the adjacent Trondheim fjord approximately 300 m from land at 40–65 m depth. Sedimented sludge is thickened and pasteurized at 70 °C for a minimum of one hour, followed by anaerobic digestion at 35 °C for 20–25 days. Together, NO1 and NO2 produce around 8 t digested and dewatered sludge annually, which can be used as agricultural fertilizer and in soil-mixes for parks and gardens, given compliance with quality standards regarding contents of PTMs and microorganisms (Gjødselvareforskriften, 2025).

GR1 is a secondary treatment facility with a capacity of 215,000 PE and treating approximately 6 Mm^3^ of wastewater annually. The treatment comprises initial screening of large particles, grease-and grit removal, chemically enhanced primary sedimentation, an activated sludge (A^2^O) process, followed by secondary sedimentation and disinfection. Disinfection by chlorination and UV irradiation is performed in separate lines, and this study only includes wastewater treated with chlorination. During treatment, a removal of ≥ 88% of BOD5, ≥ 86% of SS, ≥ 75% of the nitrogen, and ≥ 74% of the phosphorus is achieved (**Supplementary Table 1**). Treated wastewater is discharged at a depth of 54 m approximately 600 m from land. The sludge is thickened and undergoes anaerobic digestion at 33-35 °C. GR1 produces around 1,200 t digested and dewatered sludge yearly, which is landfilled with household waste.

### 2.2 Collection of wastewater and digested sludge samples

Inlet (untreated) and outlet (treated) wastewater, and digested sludge samples (indicated with green circles in **Figure 1**) were collected at three time points within a year (June, August, and October 2024 for NO1 and NO2; September and November 2024, and January 2025 for GR1). In total, 84 samples were collected, comprising 9 samples for each sample type at each WWTP (**Supplementary Table 2**). At the three time points, 24-hour composite samples of inlet and outlet wastewater were collected on three consecutive days by refrigerated automatic samplers. Samples from NO1 and NO2 were volume-proportional with a final volume of 17.5 L, while samples from GR1 were time-proportional and composed of 24 × 100 mL samples. To account for sludge digestion time, grab samples of digested and dewatered sludge were collected for three consecutive days, 20–25 days after collection of wastewater samples. All samples were kept at 4 °C until processing, within 24 hours of sample collection.

### 2.3 Quantification of antibiotic residues in wastewater and digested sludge

A total of 21 antibiotics were quantified in wastewater and digested sludge samples (**Table 1**). While all samples from NO1 and NO2 were analyzed separately (n = 9 for inlet, outlet, and digested sludge), samples from GR1 were pooled for each timepoint (n = 3 for inlet, outlet, and digested sludge). Antibiotics were selected for quantification based on sales in Norway in 2022 (NORM/NORM-VET, 2023), usage at the hospital connected to WWTP NO1 in 2022, partly self-reported consumption in Greece (Maltezou et al., 2016), previous reports on pharmaceuticals in Norwegian and Greek wastewater and digested sludge (Papageorgiou et al., 2016; Blytt and Stang, 2018; Grung et al., 2023), as well as their relevance according to the AWaRe classifications by WHO (WHO, 2023).

**Table 1:** Antibiotics quantified in samples of wastewater and digested sludge.

Antibiotic class	Antibiotics
Penicillin β-lactamases	Ampicillin, mecillinam, amoxicillin, benzylpenicillin, penicillin V, piperacillin, cloxacillin, dicloxacillin
Cephalosporins	Cefazolin, cefotaxime
Carbapenems	Meropenem
Fluoroquinolones	Ciprofloxacin, levofloxacin
Glycopeptides	Vancomycin
Lincosamides	Clindamycin
Macrolides	Azithromycin, erythromycin
Sulfonamides	Sulfapyridine, sulfamethoxazole
Tetracyclines	Tetracycline
Diaminopyrimidindes	Trimethoprim

Inlet and outlet wastewater samples were filtered through sterile 0.45 µm polyethersulfone membrane filters (Thermo Fisher Scientific) and pH adjusted to 2–3 with hydrochloric acid. Sludge samples (5 g) were extracted with methanol, which was evaporated before the extracts were re-dissolved in water. All samples (water and sludge extracts) were extracted by solid phase extraction (SPE) utilizing Oasis HLB 12 cc cartridge (Waters). In short, the cartridges were activated by 1 mL of methanol, washed with 1 mL of water before the pH adjusted sample (100 mL) was applied. The cartridge was washed with 1 mL water before the analytes were eluted with 1 mL methanol. The eluted samples were injected on an Agilent 1290 (Agilent, Santa Claria, CA, USA) ultra-high performance liquid chromatography system coupled to a Bruker Impact II (Bruker GmBH, Bremen, Germany) high resolution mass spectrometer operated in positive mode with alternating MS1 and broad-band CID fragmentation mode. The chromatographic separation was conducted by utilizing a BEH C18, 2.1 × 50 mm column (Waters) eluted with a gradient of water and acetonitrile, both containing 0.1% formic acid. Calibration curves ranging from 0.00227 µM to 2.27 µM were created by mixing the different antibiotics and processing the calibration mix within the same pipeline as the samples, including the SPE extraction. Quantification was done by TASQ (v. 2026.1.4; Bruker Daltonics GmBH) utilizing the most abundant ions from either MS1 or bbCID, as well as ion ratios for the most abundant ions for quality assurance. Details regarding the chromatographic and mass spectrometric methods and data handling can be found in the **Supplementary Methods**.

### 2.4 Quantification of potentially toxic metals and biochemical parameters in wastewater and digested sludge

Eight PTMs (arsenic (As), lead (Pb), cadmium (Cd), copper (Cu), chromium (Cr), mercury (Hg), nickel (Ni), and zinc (Zn)) were quantified in five or three day composites of inlet (n = 9), outlet (n = 9), and digested sludge (n = 9) samples, while biochemical parameters (total suspended solids (TSS), chemical oxygen demand (COD), BOD5, total nitrogen (TN), and total phosphorus (TP)), were quantified in one single 24-hr composite sample for each time point. PTMs and biochemical data for NO1 and NO2 were obtained from routine analysis data provided by the WWTPs (analyzed by Eurofins, Moss, Norway) or analyzed directly by Eurofins (Moss, Norway). Biochemical data for GR1 wastewater was collected from the National Database for monitoring the operation of wastewater treatment plants in Greece (analytical procedures unavailable; Ministry of Environment and Energy of Greece, n.d.), while PTMs were quantified by Eurofins (Moss, Norway and Athens, Greece). Quantification of PTMs was performed using inductively coupled plasma mass spectrometry (ICP-MS) based on ISO 17294-2:2016 and ISO 17294-2:2023 (ISO, 2016, 2023). Cold vapor atomic fluorescence spectroscopy (CVA-AFS) based on ISO 17852:2008 (ISO, 2008), was used for quantification of Hg in wastewater samples from NO1 and NO2, and GR1 at time point 1. BOD5, COD, TN, and TP were quantified in wastewater samples from NO1 and NO2 according to NS-EN ISO 5815-1, NS-ISO 15705, NS-EN ISO 11905-1, and NS-EN ISO 15681-2 (ISO, 1998, 2002, 2019b, a), while TSS was quantified by an internal method.

### 2.5 Extraction of total DNA from wastewater and digested sludge

DNA was extracted in triplicate from each inlet (n = 25), outlet (n = 25), and digested sludge (n = 25) sample to ensure sufficient DNA yield and representativeness of the samples. Each 50 mL wastewater (inlet/outlet) replicate was centrifuged at 2,000 × g and DNA subsequently extracted separately from the resulting pellet and supernatant. DNA was extracted from the pellet using the Masterpure Gram Positive DNA Purification Kit (Biosearch Technologies), following the protocol of the manufacturer, and eluted in 40 Buffer EB (10 mM Tris-Cl, pH 8.5; Qiagen). Extraction of DNA from the supernatant was performed using AMPure XP beads (Beckman Coulter). A volume of 500 µL beads was added to the supernatant and left to incubate with rotation for one hour at room temperature. The beads were then separated from the solution using a magnetic rack, and the supernatant was removed before washing twice with 80% ethanol. DNA was eluted in 40 µL Buffer EB (Qiagen). Equal volumes of DNA extracted from the pellet and supernatant were mixed and replicates were pooled.

DNA was extracted from 0.1 g of each digested sludge replicate using the Masterpure Gram Positive DNA Purification Kit. A slightly modified protocol was employed where the sample was dissolved in sufficient TE-buffer to rehydrate and cover the sample. Due to differences in moisture content, the volume varied for each sample. The following steps were performed according to the manufacturers protocol, adjusting the volumes of reagents according to the volume of TE-buffer used to dissolve the sample. DNA was eluted in 40 µL Buffer EB (Qiagen) and replicates were pooled. The quality and quantity of all extracted DNA were determined using a NanoDrop spectrophotometer (Thermo Fisher Scientific) and a Qubit 2.0 fluorometer with the dsDNA BR Assay Kit (Thermo Fisher Scientific), respectively.

### 2.6 16S rRNA gene sequencing and taxonomic classification

All extracted DNA samples were sequenced separately, except for the inlet, outlet, and sludge samples from GR1 at time point 1, which were pooled for each sample type. Sequencing libraries targeting the V3-V4 16S rRNA gene region were prepared according to the “16S Metagenomic Sequencing Library Preparation” protocol (Illumina, 2013). In brief: PCR amplicons were prepared using the locus-specific primers Bakt_341F and Bakt_805R (Herlemann et al., 2011) with Illumina overhang adapters (forward: 5’-TCGTCGGCAGCGTCAGATGTGTATAAGAGACAG-CCTACGGGNGGCWGCAG-3’, reverse: 5’-GTCTCGTGGGCTCGGAGATGTGTATAAGAGACAG-GACTACHVGGGTATCTAATCC-3’; Eurofins Genomics), followed by indexing using the Nextera XT Index kit (Illumina). Purified indexed amplicons were sequenced on an Illumina MiSeq sequencer (Illumina, Inc., Eindhoven, Netherlands), using the MiSeq Reagent Kit V3 (Illumina) with a 2 × 300 bp paired-end configuration. Reads were basecalled and demultiplexed in the Local Run Manager software (v. 3.1; Illumina), followed by bioinformatic processing in CLC Genomics Workbench (v. 25.0; Qiagen), with the CLC Microbial Genomics Module plugin (v. 25.1, Qiagen). Reads were trimmed for adapters and low-quality nucleotides using “Trim Reads 3.0” with a quality limit of 0.05 and filtered using “Filter Samples Based on number of Reads 1.04”, removing samples with less than 100 reads and less reads than 1% of the median. Chimeric sequences were removed and reads were clustered at a level of 97% similarity using the “OTU Clustering 2.7” tool, followed by normalization by copy number using “Normalize OTU Table by Copy Number (beta) 1.0” with the PICRUSt2 Multiplication Table (Douglas et al., 2020). Taxonomic assignment was performed using the RefSeq Prokaryotic 16S database (v. 1.0; Goldfarb et al., 2024) as a reference. Downstream analyses were performed in R (v. 4.5.0; R Core Team, 2025) using the phyloseq (v. 1.34.0; McMurdie and Holmes, 2013) and vegan (v. 2.6-10; Oksanen et al., 2025) packages. Alpha diversities were calculated from OTU tables rarefied to a sequencing depth of 3,292 reads, corresponding to the sample with the fewest reads. Rarefaction was repeated 100 times, and the mean value was reported for each alpha diversity metric. Beta diversities were based on relative abundances to account for uneven sequencing depth while avoiding the data-loss associated with rarefaction.

### 2.7 Antimicrobial resistance gene screening by hybrid capture-based sequencing

ARG composition was analyzed using the QIASeq xHYB AMR Panel (Qiagen). This panel is used for hybrid capture-based enrichment of ARGs in sequencing libraries, prior to next generation sequencing, to detect and quantify 2,786 genetic targets (**Supplementary Table 3**; Sekizuka et al., 2023). Prior to analysis, DNA extracted from the three consecutive sampling days (Section 2.5) were pooled to give one sample per time point for each of the sample types, in total 27 samples. All steps in the procedure were performed according to the manufacturers protocol. Metagenomic libraries were constructed by fragmentation, followed by adapter ligation and PCR amplification using the FX DNA Library kit (Qiagen). Purified libraries were pooled and denatured before mixing with the probes. Probes and DNA were hybridized overnight before removing non-hybridized DNA and unbound probes. Finally, bound fragments were resuspended in Hyb Elute buffer. The final libraries were sequenced on an Illumina MiSeq sequencer (Illumina, Inc., Eindhoven, Netherlands) running the MiSeq Reagent Kit V2 (Illumina) in a 2 × 150 bp paired-end configuration. Basecalling, demultiplexing, and read trimming were performed as described in Section 2.6. Resistance genes were quantified in CLC Genomics Workbench v. 26.0 (Qiagen) with the “Find Resistance with ShortBRED” tool (v. 1.5) using default settings (e-value = 0.00001, percent identity = 0.95, minimum alignment length = 0.95, minimum read length = 90.0) and the QMI-AR Peptide Marker Database (v. 2021-08). Downstream analyses were performed in R. Genes were classified according to ARO drug classes (antibiotic resistance classes; Alcock et al., 2020), and genes solely classified in the “disinfecting agents and antiseptics” antibiotic resistance class were removed before calculating relative abundances and further downstream analyses.

### 2.8 Enumeration and isolation of fecal and environmental indicator bacteria from wastewater and digested sludge

Wastewater samples (inlet/outlet) were serially diluted in peptone water (8.5 g/L NaCl; 1 g/L neutralized peptone) and 50 mL of the dilutions were filtered through 0.45 µm cellulose nitrate membrane filters (Thermo Fisher Scientific). Filters were placed onto selective agars for enumeration and isolation of indicator bacteria. Presumptive coliform bacteria, including *E. coli* were enumerated and isolated on RAPID’*E. coli* 2 Agar supplemented with RAPID’*E. coli* 2 supplement (Bio-Rad Laboratories) and Harlequin *E. coli*/Coliform Agar (Neogen). Presumptive *Enterococcus* were enumerated and isolated on *Enterococcus* Selective Agar (Millipore). Presumptive *Vibrio* were enumerated and isolated on Thiosulfate Citrate Bile Sucrose (TCBS) Agar (Millipore) supplemented with marine broth 2216 (Millipore). H_2_S producing bacteria (here assumed to be *Aeromonas*; NMKL no. 184, 2006) were enumerated and isolated as black colonies on Lyngby Iron Agar (Oxoid) supplemented with 0.04% L-cysteine (Sigma-Aldrich). Presumptive *Pseudomonas* were enumerated and isolated on Pseudomonas Agar Base supplemented with *Pseudomonas* CFC Supplement (CFC; Oxoid). RAPID’*E. coli* 2, Harlequin *E. coli*/Coliform, and *Enterococcus* Selective Agar plates were incubated at 37 °C for 24 and 46 hours, respectively. Presumptive fecal enterococci were further confirmed by transferring the filter to Bile-Aesculin Agar (Oxoid) and incubating at 44 °C for 2 hours. TCBS, Lyngby Iron Agar, and CFC agar plates were incubated at 25 °C for 46 hours. Sludge samples were prepared by homogenizing 20 g sludge together with 180 mL peptone water (1:10 dilution) in a stomacher homogenizer for 1 min. Serial dilutions were prepared in peptone water, plated on selective agars, and incubated as described for wastewater samples. Typical colonies were isolated based on color and morphology, and re-streaked onto their respective selective agar to ensure purity before being preserved in tryptic soy broth with 20% glycerol at -80 °C.

### 2.9 Taxonomic classification of indicator bacteria

Classification of isolates was performed based on the V3-V9 region of the 16S rRNA gene. A colony was picked from a freshly incubated culture and lysed by heating in nuclease-free water for five minutes at 99 °C. The suspension was used directly in the PCR-amplification using DreamTaq MasterMix (Thermo Fisher Scientific) and the primer pair 338F (5’-ACTCCTACGGGAGGCAGCAG-3’) and 1492R (5’-GGTTACCTTGTTACGACTT-3’) (Muyzer et al., 1993; Turner et al., 1999), obtained from Sigma-Aldrich, and the following conditions: Initial denaturation at 95 °C for 2 min., followed by 30 cycles of denaturation at 95 °C for 30 s., annealing at 58 °C for 30 s., and extension at 71 °C for 1 min., and final extension at 72 °C for 5 min. PCR-products were purified with ExoSAP-IT (Thermo Fisher Scientific) and prepared for Sanger sequencing by mixing 5 µL DNA (25–50 ng/µL) and 5 µL of the primer 338F (5 ng/µL). Sanger sequencing was performed by Eurofins Genomics (Ebersberg, Germany). Sequences were aligned to the NCBI 16S ribosomal RNA (Bacteria and Archaea) database (downloaded 16.01.2025) using BLAST+ (v. 2.16.0; Camacho et al., 2009). Isolates from RAPID’*E. coli* 2 agar, classified as *Escherichia marmotae* and *Escherichia fergusonii* were assumed to be *Escherichia coli* based on whole genome sequencing of representative isolates (unpublished data).

### 2.10 Antibiotic susceptibility testing of indicator bacteria

#### 2.10.1 Robotic high-throughput antimicrobial susceptibility testing

Antimicrobial susceptibility testing (AST) of isolates from NO1 and NO2, classified as coliform bacteria, *E. coli, Aeromonas*, and *Pseudomonas* by 16S rRNA gene sequencing (**Supplementary Table 4**), was performed against 10 antibiotic compounds (**Table 2**). Each strain was tested against three concentrations of each antibiotic, and an additional well without antibiotics was included as a growth control. Reference strains (*E. coli* ATCC 25922, NCTC 13846, CCUG 59342, and CCUG 59351, and *Pseudomonas aeruginosa* ATCC 27853) were included for quality control. Prior to testing, Nunc flat-bottom 384-well microplates (Thermo Fisher Scientific) were prefilled with antibiotics (sourced from Sigma-Aldrich), in nanoliter volumes, by an Echo 650 series acoustic liquid handler (Beckman Coulter Inc., Brea, CA, USA). Filled plates were sealed and stored at -20 °C until usage.

**Table 2:** Antibiotics included in antimicrobial susceptibility testing.

Antibiotic	Concentration coliforms (mg/L)	Concentration *Aeromonas* and *Pseudomonas* spp. (mg/L)	Solvent
Ampicillin	4, 8, 16	8, 32, 128	Water
Ciprofloxacin	0.125, 0.25, 0.50	0.25, 0.5, 1	DMSO
Colistin	1, 2, 4	2, 4, 8	Water
Ceftazidime	0.5, 2, 4	1, 4, 16	DMSO
Cefotaxime	0.5, 1, 2	8, 32, 128 (2, 8, 32 for *Aeromonas*)	Water
Gentamicin	1, 2, 4	2, 4, 16	Water
Meropenem	0.125, 0.50, 4	1, 4, 8	DMSO
Piperacillin-tazobactam*	4, 8, 16	4, 16, 128	Water
Tetracycline	4, 8, 16	0.5, 32, 128	Water
Trimethoprim-sulfamethoxazole**	1, 2, 4	0.5, 8, 64	DMSO

Final assessment concentrations and solvent used to make high-concentration stock-solutions are given.

*Concentrations of Piperacillin are given. Tazobactam was tested at a constant concentration of 4 mg/L.

**Tested in ratios of 1:19 of trimethoprim: sulfamethoxazole. Concentrations of trimethoprim are given.

The AST was performed on an Biomek i7 Automated Workstation (Beckman Coulter Inc., Brea, CA, USA). Pre-cultures were prepared by adding 50 µL of thawed cryopreserved cultures and 450 µL Tryptone Soya Broth (TSB; Oxoid) to 96 deep well V-bottom plates (Greiner Bio-One GmbH), followed by overnight incubation at 35 °C, 85% relative humidity, and 900 rpm (25 mm amplitude). *Pseudomonas* isolates with limited growth were incubated at 25 °C (**Supplementary Table 4**). Pre-cultures were diluted 1:4000 in Mueller Hinton Broth II (MHBII; Oxoid) and 37.5 µL was transferred, in triplicate, to the 384 well plates prefilled with antibiotics. Plates were covered with lids, packed in plastic bags, and incubated for 20–24 hours. Incubation was carried out statically for 20–22 hours in the same conditions as the pre-cultures.

Following incubation, optical density at 600 nm (OD_600_) was determined using a Tecan Spark microplate reader (Tecan, Männedorf, Switzerland). The lowest concentration of antibiotic resulting in a growth reduction of ≥ 90% compared to the growth control was noted as the operational MIC. Susceptibility was determined based on epidemiological cut-off (ECOFF) values (Kahlmeter and Turnidge, 2022; EUCAST, n.d). If species-specific ECOFFs were not available, the ECOFF of species within the same genus was used as a proxy, and when this was not available, clinical breakpoints were used instead. To retain a conservative approach the S/I clinical breakpoints were used. See **Supplementary Table 5** for cut-off values.

#### 2.10.2 Manual antibiotic susceptibility testing

Antibiotic susceptibility of isolates from GR1, classified as coliform bacteria, *E. coli, Aeromonas*, and *Pseudomonas* by 16S rRNA gene sequencing (**Supplementary Table 4**), was assessed using Sensititre EUVSEC3 AST plates (Thermo Fisher Scientific), while the antibiotic susceptibility of isolates classified as *Enterococcus* spp. from NO1 and NO2 (**Supplementary Table 4**) was assessed using Sensititre EUVENC AST plates (Thermo Fisher Scientific). Testing was performed according to the protocol outlined by the manufacturer. Briefly, colonies grown overnight were dissolved in sterile de-ionized water to a density of 0.5 McFarland. For *Enterococcus* isolates, 30 µL of the suspension was transferred to 11 mL of MHBII, while for all other isolates, 10 µL was transferred. Aliquots of 50 µL were added to each well of the appropriate plate and the plate was sealed before incubation at 35 °C (or 25 °C) 20–24 hours. Results were read using the Sensititre Manual Viewbox (Thermo Scientific) and the MIC was determined as the lowest concentration of the antibiotic inhibiting visible growth (80-90% of growth control for linezolid). Susceptibility was determined as described in Section 2.10.1. For the AST of *Enterococcus* spp., *Enterococcus faecalis* ATCC 29212 was included as a quality control. To ensure comparability between the AST methods used at NO1 and NO2, and GR1, a subset of isolates (n = 19) was tested on both the robotic high-throughput screening platform and by using the Sensititre EUVSEC3 plates. Classification as susceptible or non-susceptible was consistent for ≥ 84% of the isolates for all antibiotics (**Supplementary Table 6**).

### 2.11 Statistical analysis

Statistical analyses were performed in R (v. 4.5.0; R Core Team, 2025). Alpha diversity parameters were compared by analysis of variance (ANOVA) with Tukey’s *post-hoc* test using the functions *aov* and *tukeyHSD* of the stats package. Normality of residuals and homogeneity of variance were assessed by the Shapiro-Wilk test and Levene’s test with a significance level of 0.05, respectively. Differences in beta diversity were investigated by permutational multivariate analysis of variance (PERMANOVA), using the functions *adonis2* of the vegan package and *pairwise.adonis* (Martinez, 2020). In both cases, location, sample type, and time point were included as main effects and all two-way interactions were included. The three-way interaction was omitted since it did not significantly improve the model. Wilcoxon signed rank tests and Kruskal-Wallis tests with Dunn’s *post-hoc* analysis were applied to assess reduction in CFU, antibiotic residues, and PTMs by treatment and between-plant differences. The fit of antibiotic concentrations to Bray-Curtis Non-metric Multidimensional Scaling (NMDS) ordinations was tested using the *envfit* function in vegan, which performs a permutation-based significance test, on Z-normalized data. Procrustes analysis, using the function *Procrustes* from the vegan package was used to assess the correlation between bacterial community and ARG ordinations. Statistical significance of the correlation was assessed by permutation test using *protest.* Differences in reduced susceptibility rates were assessed by Fisher’s exact test using the function *fisher.test*. When multiple tests were performed, p-values were adjusted using the Benjamini-Hochberg method.

## 3 Results and discussion

### 3.1 Concentrations of antibiotics in wastewater and digested sludge vary across wastewater treatment plants

**Figure 2:**
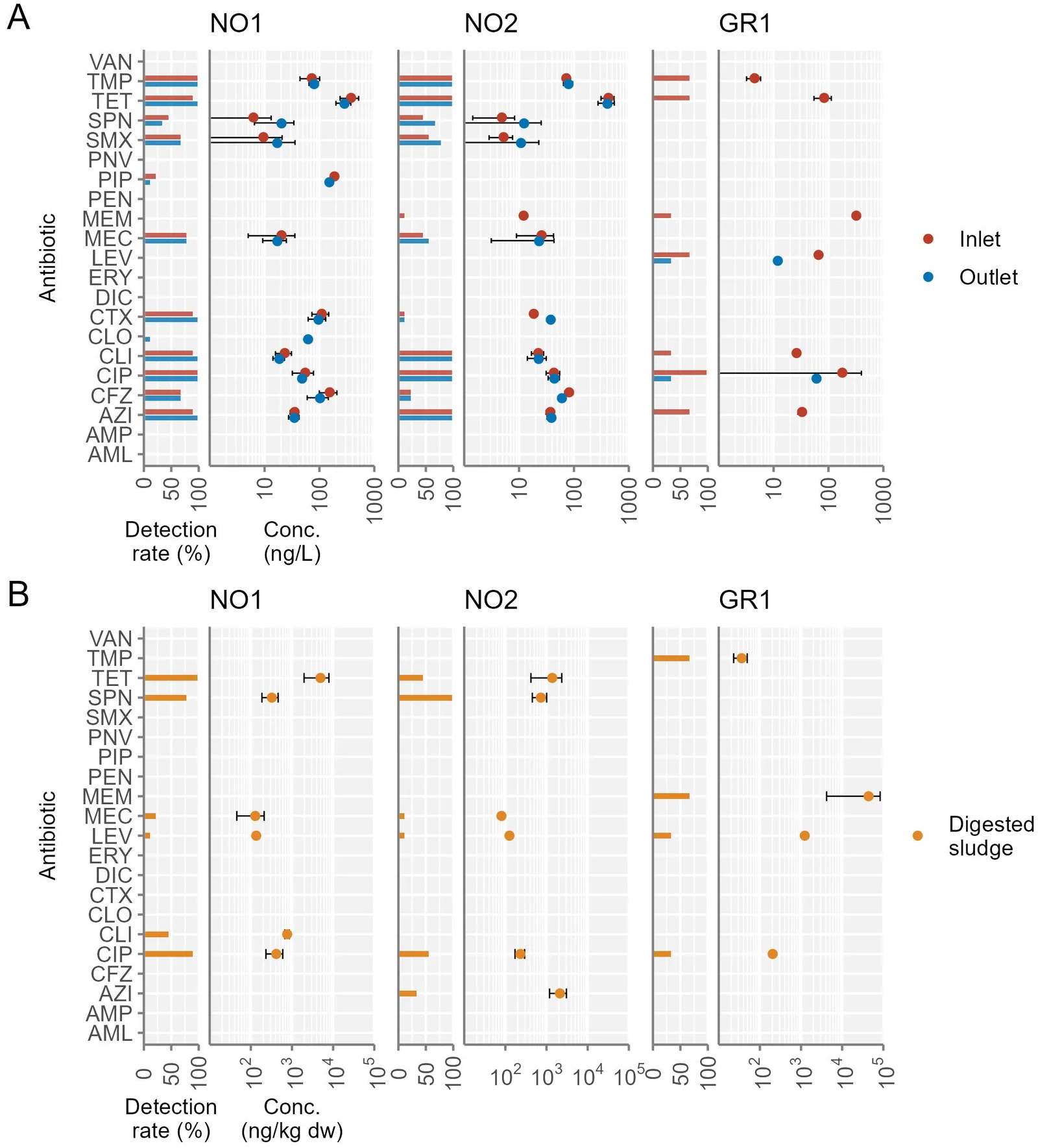
Detection rates and concentrations of antibiotic residues in samples from NO1, NO2 and GR1. **(A)** Inlet (n = 21) and outlet (n = 21), **(B)** digested sludge (n = 21). Bars represent detection rate across samples and points are arithmetic means of samples with concentration > 0. Error bars represent standard deviations (SD) and were calculated for antibiotics detected in ≥ 2 samples. VAN, Vancomycin; TMP, Trimethoprim; TET, Tetracycline; SPN, Sulfapyridine; SMX, Sulfamethoxazole; PNV, Penicillin V; PIP, Piperacillin; PEN, Penicillin G; MEM, Meropenem; MEC, Mecillinam; LEV, Levofloxacin; ERY, Erythromycin; DIC, Dicloxacillin; CTX, Cefotaxime; CLO, Cloxacillin; CLI, Clindamycin; CIP, Ciprofloxacin; CFZ, Cefazolin; AZI, Azithromycin; AMP, Ampicillin; AML, Amoxicillin.

Antibiotic residues were quantified in inlet, outlet, and digested sludge samples collected from the three WWTPs. Of the 21 antibiotics investigated, 13 were detected in at least one sample (**Figure 2**). The most frequently detected antibiotic was ciprofloxacin, which was detected in 100% of inlet samples, 90% of outlet samples, and 67% of all digested sludge samples. In total, seven antibiotics were detected in at least one wastewater sample at GR1, as compared to 12 and 11 at NO1 and NO2, respectively. While several antibiotics (azithromycin, clindamycin, ciprofloxacin, tetracycline, and trimethoprim) were detected in 89-100% of the inlet samples from NO1 and NO2, detected antibiotics at GR1 commonly occurred in only 2/3 (67%) of the pooled inlet samples. However, samples from GR1 were pooled at each time point, potentially masking temporal variation in occurrences and concentrations. Consequently, the reported detection frequencies, variation in concentrations, and comparisons with NO1 and NO2 should be interpreted in light of this. Even so, the concentration of ciprofloxacin in the inlet of GR1 ranged from 10 ng/L up to 427 ng/L, which is approximately 4 and 7 times higher than the highest concentrations measured in the inlets of NO1 and NO2, respectively. The PNEC for selection (64 ng/L; Bengtsson-Palme and Larsson, 2016) was exceeded in 2/3 of the pooled inlet samples from GR1. Furthermore, meropenem was detected at concentrations exceeding the suggested PNEC of 64 ng/L by more than tenfold in the inlet of GR1 at time point 3. While exceedance of the PNECs does not constitute evidence of selection, it indicates that concentrations were within ranges where potential effects on microbial communities warrant consideration.

Piperacillin, which is commonly used in combination with the β-lactamase inhibitor tazobactam, was detected only in inlet and outlet at NO1, while the 3^rd^ generation cephalosporin cefotaxime and 1^st^ generation cephalosporin cefazolin were more frequently detected at NO1 than NO2, and at significantly higher concentrations (Dunn’s test, p < 0.01). Cefotaxime was occasionally detected in concentrations above the PNEC of 125 ng/L in both inlet and outlet samples from NO1. This reflects antibiotic consumption patterns at the connected hospital and is therefore likely attributable to hospital wastewater input. Considering the increasing incidence of bloodstream infections with piperacillin-tazobactam resistant *P. aeruginosa* and 3^rd^ generation cephalosporin resistant *Klebsiella pneumoniae* (ECDC, 2024b), these observations are concerning.

During treatment, concentrations of all antibiotics except the fluoroquinolones ciprofloxacin and levofloxacin decreased to below the detection limit in the pooled outlet samples from GR1. In contrast, the decrease in concentration from inlet to outlet was limited at NO1 and NO2 (paired Wilcoxon, p > 0.05). The efficient removal observed at GR1 is likely attributable to the activated sludge process, which generally contributes more to removal than primary settling (Watkinson et al., 2007), the main treatment applied at NO1 and NO2. In the case of sulfonamides, concentrations consistently increased from inlet to outlet at NO1 and NO2. This has also previously been observed in primary wastewater treatment processes and was associated with transformation of conjugated metabolites back to the parent antibiotic compound (Göbel et al., 2007). In most cases, concentrations of the quantified antibiotics in effluents were within the ranges previously reported for European WWTPs (Rodriguez-Mozaz et al., 2020), except for tetracycline where we found higher concentrations, as well as the detection of cefotaxime and cefazolin, which were not found in the previous study. Since they performed their investigations in 2015 and 2016, the total consumption of tetracycline and cefazolin in Norway, and the usage of 3^rd^ generation cephalosporins in Norwegian hospitals have increased (NORM/NORM-VET, 2023). Together with the differences in treatment technologies, this may partly explain the discrepancies.

Digested sludge represents an additional environmental discharge pathway, and seven of the 21 antibiotics were detected in at least one sample (**Figure 2**). The most frequently detected antibiotic in digested sludge at NO1 and NO2 was sulfapyridine (100 and 78%). While sulfapyridine is rarely used for antibacterial purposes in Norway, instead being primarily used as an anti-inflammatory agent, it is still of high relevance in the context of AMR. Meropenem and trimethoprim were the most frequently detected antibiotics in sludge at GR1 (2/3 pooled samples), with meropenem having the highest mean concentration (44 ± 40 µg/kg d.w.). Ciprofloxacin, detected in digested sludge at all three WWTPs, and tetracycline, detected at NO1 and NO2, have long half-lives in biosolid-amended soils (2310 and 578 days, respectively; Walters et al., 2010). Accordingly, soil application or landfilling of digested sludge from all three WWTPs treatment plants may allow prolonged exposure and raises concerns regarding the potential for selection of ARB and ARG.

### 3.2 Potentially toxic metals are present in wastewater and digested sludge at all wastewater treatment plants

**Figure 3:**
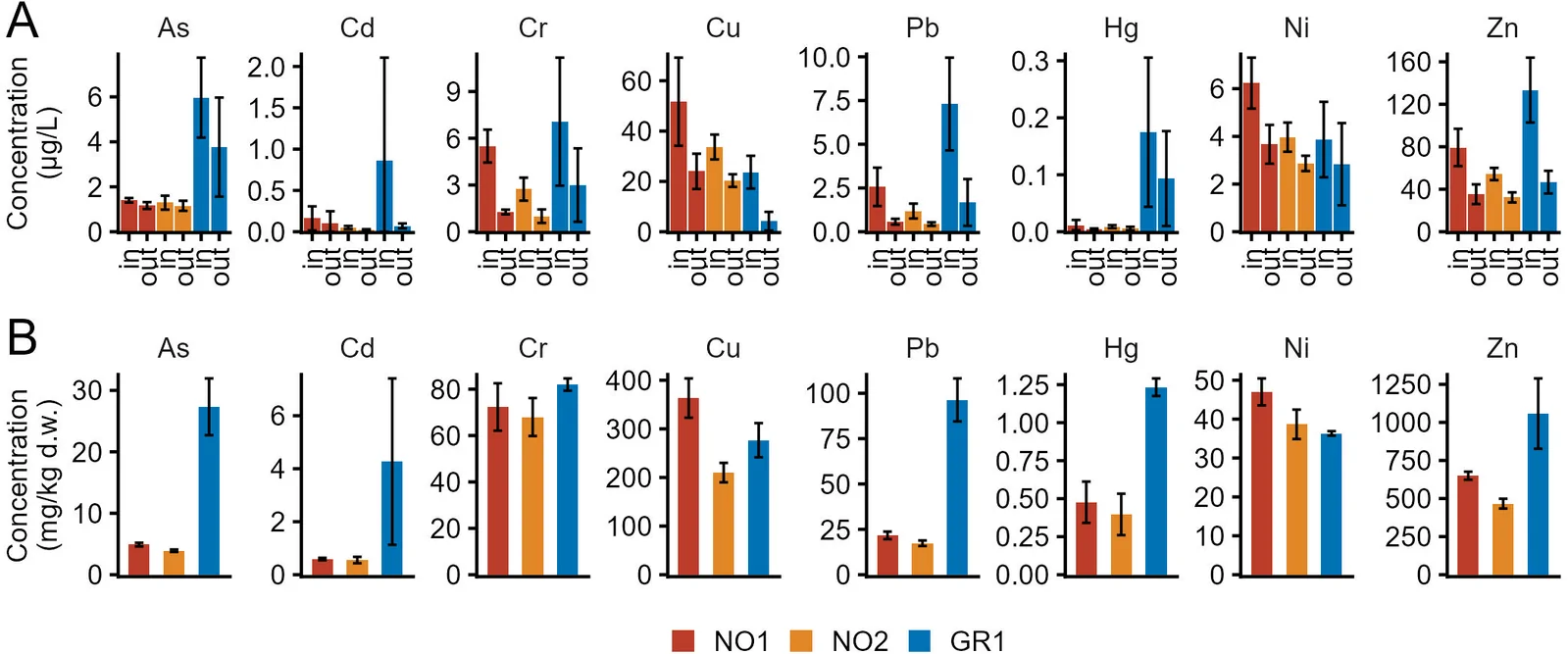
Concentrations of PTMs in samples from NO1, NO2, and GR1. **(A)** inlet (in, n = 9) and outlet (out, n = 9), **(B)** digested sludge (n = 9). Bars represent the arithmetic mean of three measurements within a year, and error bars represent ± SD. Sludge concentrations are given on a dry weight basis. As, Arsenic; Cd, Cadmium; Cr, Chromium; Cu, Copper; Pb, Lead; Hg, Mercury; Ni, Nickel; Zn, Zinc.

To investigate differences in PTM concentrations across WWTPs and sample types, eight PTMs were quantified in weekly composite samples at the three time points for inlet, outlet, and digested sludge. Across wastewater samples (inlet and outlet), mean concentrations were highest at GR1 for all metals, except for Cu and Ni, which had higher concentrations at NO1 (**Figure 3**). Statistical significance was, however, limited to As and Hg (Dunn’s test, p < 0.05). No significant mean reduction from inlet to outlet was observed, despite high removal efficiencies being reported for several of the PTMs also in primary treatment facilities (Hargreaves et al., 2018). Given the limited number of samples per sample type at each WWTP (n = 3), the ability to detect differences was likely constrained by low statistical power. Both inlet and outlet concentrations consistently exceeded the proposed minimal co-selective concentration (MSC) for Cu, Ni, Pb, and Zn at all three WWTPs (Seiler and Berendonk, 2012). At GR1, the MSC for Cd and Hg (Seiler and Berendonk, 2012; Arya et al., 2021) was also exceeded. In addition to their potential role as selective agents during wastewater treatment, PTMs can accumulate in the environment and impact downstream microbial communities (Chen et al., 2022).

Overall, concentrations of PTMs in digested sludge mirrored those in wastewater, and significantly higher mean concentrations were found for As, Pb, Hg, and Zn at GR1 than NO2 (Dunn’s test, p < 0.05). Cu and Ni were found in highest concentrations in digested sludge at NO1 with Cu concentrations also being significantly higher at NO1 compared to NO2 (Dunn’s test, p < 0.05). While there are limited data on MSC for PTMs in digested sludge, correlations between metal concentrations and the abundance of ARGs have been found for Ni, Cu, and Cr in soil within the concentration ranges detected in this study (Knapp et al., 2011). Furthermore, PTM contamination is associated with changes in bacterial community composition (Liu et al., 2024), suggesting that PTMs should be assessed alongside ARG and ARB when evaluating the potential for selection and dissemination of AMR in wastewater, digested sludge, and downstream environments.

### 3.3 Microbiome diversity is shaped by location, wastewater treatment and time point of sampling

**Figure 4:**
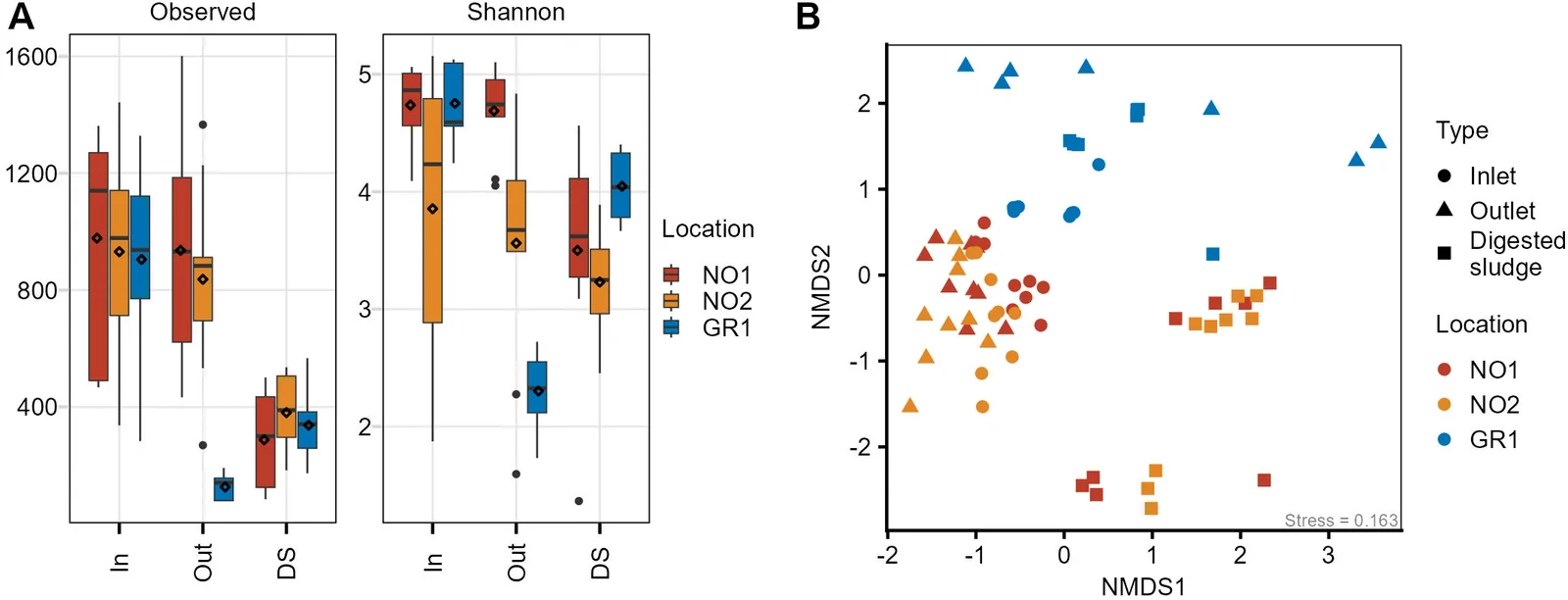
Alpha and beta diversities of bacterial communities in inlet, outlet, and digested sludge samples from NO1 (n = 26), NO2 (n = 27) and GR1 (n = 21). **(A)** Observed richness and Shannon diversity index were calculated from rarefied data. Boxes represent the interquartile range (IQR), and the line represent the median. Outliers are marked by dots and means are marked by diamonds. **(B)** NMDS ordination based on Bray-Curtis distances. Distances were calculated using relative abundances. In, inlet; Out, outlet; DS, digested sludge.

The bacterial communities of wastewater (inlet, outlet) and digested sludge samples were investigated based on 16S rRNA gene sequencing, which yielded a total of 6,131 unique Operational Taxonomic Units (OTUs), of which 96.5% were classified as bacteria. Rarefaction curves did not fully plateau for all samples but approached saturation (**Supplementary Figure 1**), suggesting that the main community structures were captured. Alpha diversities varied across locations, sample types, and time points (**Figure 4**), and ANOVA was used to discern the effects of these variables. The models explained 89 and 82% of the total variance for observed richness and Shannon diversity index, respectively. Location, sample type, and time point had significant effects on both measures (ANOVA, p < 0.05). While the observed richness was most affected by sample type (R^2^ = 0.30), the Shannon diversity index was strongly affected by both location (R^2^ = 0.14) and sample type (R^2^ = 0.13). There were also significant interactions between location, sample type, and time point (p < 0.05), indicating that the specific combination of factors is important in determining the alpha diversity.

Both observed richness and the Shannon diversity index were significantly lower in the inlet of NO2 compared to NO1 and GR1 (Tukey’s, p < 0.05), while there was no difference between the inlets of NO1 and GR1. Inlet samples at NO2 additionally exhibited highly variable observed richness and Shannon index across time points, which could be attributed to low richness at time point 1. This coincided with a low inflow from sewers resulting from dry weather. The dry weather may have caused a relatively low input of environmental bacteria associated with urban run-off and other environmental sources, resulting in a low observed richness. The variation with time point decreased from inlet to outlet, suggesting that the sedimentation process reduced the temporal variation, potentially by a process-related shaping of the community (Riisgaard-Jensen et al., 2023). No significant difference in observed richness and Shannon diversity index was found between the inlet and outlet at NO1 or NO2, while both the observed richness and Shannon diversity index decreased from inlet to outlet at GR1 (p < 0.01). Given that GR1 has additional treatment steps, including activated sludge and disinfection, this was expected.

Digested sludge communities exhibited similar richness across WWTPs, but were generally less rich than their corresponding wastewater inlet communities (p < 0.01), probably due to taxa filtering in the transition from an aerobic water environment to the anaerobic sludge, as previously indicated (Kirkegaard et al., 2017). Despite performing pasteurization before sludge digestion at NO1 and NO2, only digested sludge at NO2 exhibited lower diversity than GR1 (p < 0.05), indicating that the pasteurization processes may affect the communities at NO1 and NO2 differently.

The between-sample diversity was analyzed based on Bray-Curtis distances, and NMDS showed that samples largely clustered according to location and sample type (**Figure 4**). PERMANOVA, which explained 74% the total variance, confirmed the dependence of community composition on both location (R^2^ = 0.13, p < 0.05) and sample type (R^2^ = 0.19, p < 0.05). Additionally, time point was found to be significant (R^2^ = 0.09, p < 0.05). As observed for alpha diversity, there were significant interactions between factors. While this may partly be explained by differences in wastewater treatment processes, there were also significant differences between the inlet communities, with the composition of communities at GR1 differing significantly from NO1 and NO2 (p < 0.01). Thus, factors outside of the WWTP may also have contributed to the observed community compositions. Although not investigated in this study, several factors have been associated with wastewater bacterial community composition, including biochemical parameters, sewer microbial communities, and air temperature (Newton et al., 2015; Guo et al., 2019; Riisgaard-Jensen et al., 2025). Additionally, the time-dependence is consistent with the previously observed seasonality of wastewater bacterial communities (LaMartina et al., 2021).

Correlations between bacterial community composition and several antibiotic classes in treated wastewater have previously been demonstrated (Novo et al., 2013). In line with this, the Bray-Curtis ordination was found to correlate significantly (p < 0.05) with concentrations of several classes of antibiotics targeting different cellular targets (azithromycin, cefotaxime, sulfamethoxazole, sulfapyridine, ciprofloxacin, tetracycline, and trimethoprim), suggesting that these antibiotics may be associated with the observed differences in community composition. Although it was not possible to correlate metal concentrations to the ordinations due to the pooling of samples, the significant differences in concentrations of As and Hg in wastewater at NO1 and NO2, compared to GR1 also coincided with differences in community composition.

### 3.4 Taxonomic profiling reveals dominant bacterial taxa and treatment-driven shifts

**Figure 5:**
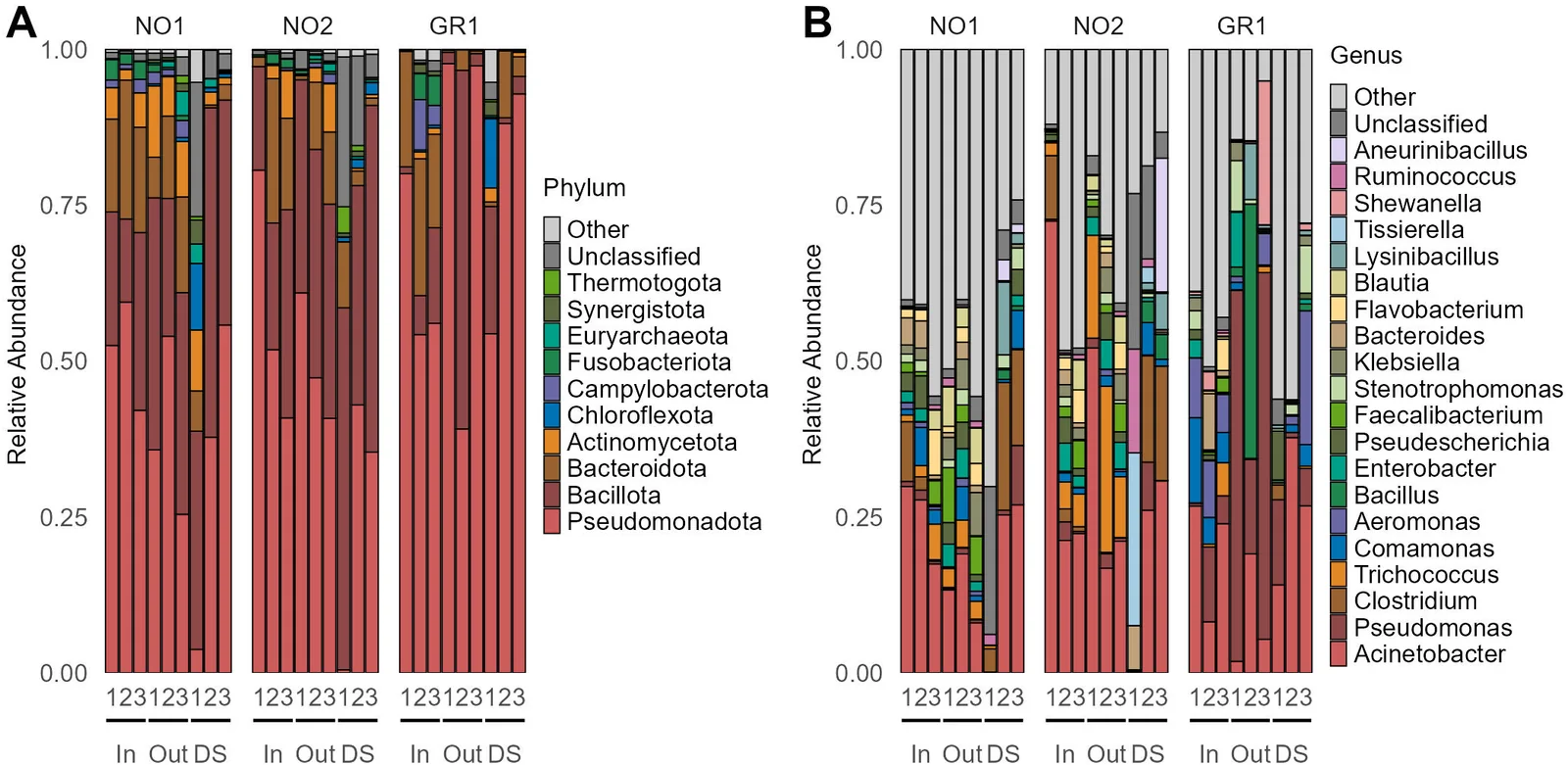
Relative abundances of the overall **(A)** top 10 phyla and **(B)** top 20 genera in inlet, outlet, and digested sludge samples from the three WWTPs. In, inlet; Out, outlet; DS, digested sludge. 1, 2, and 3 represent the three sampling timepoints and relative abundances represent the mean of three replicates for each timepoint. Unclassified taxa were included for comparison.

Alpha and beta diversity analyses revealed significant differences in community diversity and composition across WWTPs, sample types, and time points. To get an understanding of the underlying taxonomy, the relative abundances of taxa at phylum and genus level were investigated (**Figure 5**, **Supplementary Table 7**). The dominant phylum was Pseudomonadota (previously Proteobacteria, mean relative abundance of 0.50 ± 0.27), followed by Bacillota (previously Firmicutes, 0.29 ± 0.21) and Bacteroidota (0.10 ± 0.08), agreeing with previous findings (Numberger et al., 2019). At the genus level, the dominant taxon was *Acinetobacter* (0.22 ± 0.19), typically associated with sewage rather than being human-associated and suggested to be a significant contributor to the spread of AMR due to their ability to acquire resistance determinants and survive in diverse environments (VandeWalle et al., 2012; Tobin et al., 2024).

While *Acinetobacter* was the dominant genus in wastewater communities at NO1 and NO2, *Trichococcus, Flavobacterium, Faecalibacterium, Enterobacter, Pseudescherichia, Blautia, Klebsiella*, and *Bacteroides* also exhibited mean relative abundances of ≥ 1% in inlets and outlets. Both *Klebsiella* and *Enterobacter* are members of the order *Enterobacterales*, which in combination with carbapenem resistance, is listed in WHOs priority pathogen list (WHO, 2024). After treatment, the main changes were a decrease in the relative abundance of *Acinetobacter* at NO1, and an increase in the relative abundance of *Trichococcus* accompanied by a decrease in the relative abundance of *Acinetobacter* and *Clostridium* at NO2 (**Supplementary Table 8**).

Several genera were present with a relative abundance of ≥ 1% in the inlet or outlet communities at GR1 (**Supplementary Table 7**). Of these, *Aeromonas*, *Bacteroides, Flavobacterium, Shewanella, Bacillus*, and *Lysinibacillus* did not reach 1% at NO1 and NO2. From inlet to outlet, the largest changes were an increase in the relative abundance of *Pseudomonas, Bacillus, Shewanella*, and *Lysinibacillus*, accompanied by a decrease in *Aeromonas, Comamonas* and *Acinetobacter* (**Supplementary Table 8**). The community compositions were, however, highly variable in the outlet of GR1.

Like wastewater, digested sludge communities at NO1 and NO2 were represented by a high relative abundance of *Acinetobacter.* Additionally, genera with a mean relative abundance of ≥ 10%included *Clostridium* (at both NO1 and NO2) and *Tissierella* (NO2). Distinct communities were observed at both NO1 and NO2 at timepoint 1. At NO1, this can be explained by the community being in the assembly-stage due to recent sanitation. At NO2, the corresponding wastewater samples also exhibited distinct community compositions, characterized by an overrepresentation of *Acinetobacter*. This is in agreement with low inflow of environmental bacteria (Section 3.3) and a resulting relative increase of sewage-associated bacteria such as *Acinetobacter* (VandeWalle et al., 2012).

Similar to the outlet communities, the community composition in digested sludge at GR1 was highly variable, and characterized by high relative abundances of *Acinetobacter, Aeromonas* and *Pseudomonas*. *Pseudomonas*, including pathogenic species, are known to carry resistance genes and have been suggested to be a contributor to the dissemination of AMR through wastewater (Luczkiewicz et al., 2015; Butiuc-Keul et al., 2021). Specific strains must, however, be examined for pathogenicity and resistance to determine whether these genera contribute to dissemination of AMR at GR1.

### 3.5 A wide range of resistance genes were detected across WWTPs and are released into the environment

**Figure 6:**
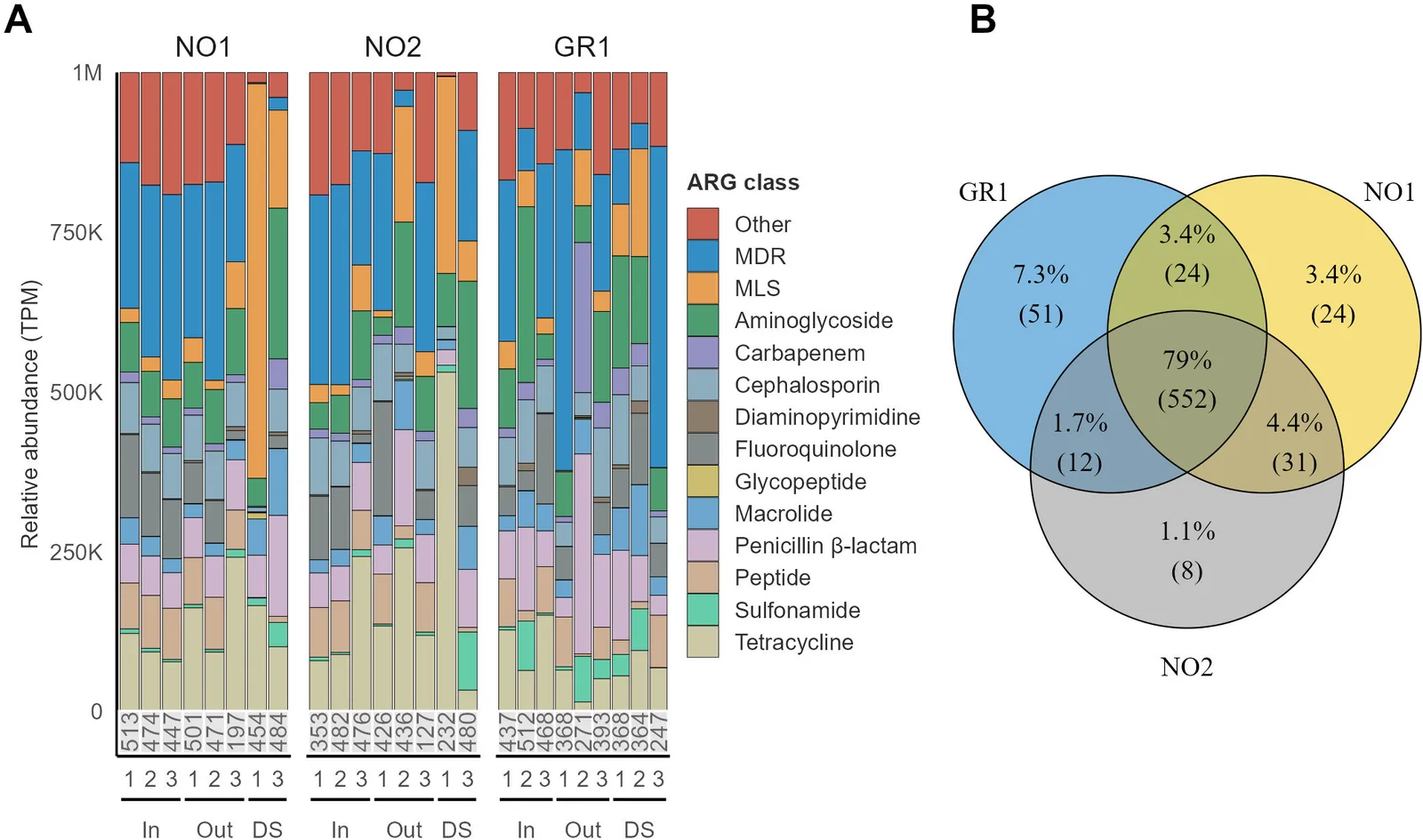
Resistomes in inlet (in, n = 9), outlet (out, n = 9), and digested sludge (DS, n = 7) samples from the three WWTPs. **(A)** Relative abundance (TPM) of antibiotic resistance classes in inlet, outlet, and digested sludge samples. **(B)** The proportion and number of ARGs shared between WWTPs and specific to one or two WWTPs. No results were obtained for DS samples from NO1 and NO2 at time point 2.

The resistomes of the inlet, outlet, and digested sludge samples from the three WWTPs were characterized by assessing the occurrence and abundance of 2,786 gene targets. The three samples from each time point were pooled prior to analysis. In total, 710 distinct genes and gene families were detected in at least one sample and the number of genes per sample varied from 127-513 (**Figure 6**). The dominant antibiotic resistance classes were multidrug resistance (MDR; mean relative abundance of 20 ± 14%), tetracycline (13 ± 10%), and aminoglycoside (10 ± 6%). A similar distribution has also been observed at a different Norwegian WWTP (Lindstedt et al., 2025). Cephalosporin (7 ± 3%) and carbapenem (3 ± 5%) resistance genes were less abundant relative to the aforementioned antibiotic resistance classes but, considering their importance in treatment of infections caused by MDR bacteria, their widespread occurrence underscores the need for surveillance, especially to detect temporal changes.

The ARG composition, summarized by antibiotic resistance class, appeared similar across the three WWTPs, with variations that could not be explained by location, sample type, or time point alone (**Figure 6**, **Supplementary Table 9**). Like previously observed (Ju et al., 2019), Procrustes analysis revealed a moderate correlation between ARG and bacterial community composition (r = 0.75, p < 0.01), suggesting that the observed variation may be associated with the bacterial communities present at the three WWTPs. ANOVA and PERMANOVA were used to assess the effects of location and sample type on the number and composition of ARGs, respectively. This revealed a significantly lower number of ARGs in digested sludge compared to inlet and outlet wastewater (p < 0.01) and significant differences in the ARG composition between sample types (R^2^ = 0.17, p < 0.05), but not between WWTPs (p = 0.37). No differences were found between the inlet and outlet resistomes (p = 0.23), while pairwise PERMANOVA showed significant differences between inlet and digested sludge (p < 0.01). The largest differences were observed for the MDR and MLS antibiotic resistance classes. This could potentially be associated with differences in community composition as digested sludge exhibited the most distinct community profiles. Digested sludge also showed a variable ARG class composition across timepoints, which coincided with the variation in community composition (**Figures 5**, **6**), further implying a correlation with community composition.

There was a considerable overlap in resistance genes detected across the three WWTPs, with 79% of all genes shared among all locations, and only 3.4, 1.1, and 7.2% of the genes were specific to NO1, NO2, and GR1, respectively (**Figure 6**, **Supplementary Table 10**). While a similar pattern was observed when comparing only inlet samples, the overlap between inlet, outlet, and digested sludge within each treatment plant was lower, with 44-66% of the genes being shared between all sample types (**Supplementary Figure 2**). The overlap between inlet and outlet at GR1 (71%) was slightly lower than at NO1 and NO2 (82-85%). Together with the non-significant differences in composition between WWTPs, this indicates that wastewater treatment has an effect comparable to or greater than geographical location. Some important genes were limited to one or two of the WWTPs. The gene *mecA* was only observed in inlet and outlet samples from NO1 and NO2, while *mecC* was observed only in the inlet of NO1. These genes confer methicillin resistance in *Staphylococcus aureus*. This pattern was surprising since the clinical prevalence of methicillin-resistant *S. aureus* (MRSA) is considerably higher in Greece than in Norway (ECDC, 2024b). Although these genes are widely associated with MRSA, they are also common in coagulase-negative staphylococci (Xu et al., 2018). Thus, the prevalence of these genes in wastewater may reflect a broader diversity of bacterial hosts than clinical MRSA alone. While most β-lactamase gene families were detected at all three locations, the highly mobile β-lactamase gene *bla*_VMB-1_, exhibiting carbapenemase-activity (Zheng et al., 2020), and *bla*_VIM_ genes were only detected at NO1 and GR1. As *bla*_VIM_ genes are frequently detected in hospital wastewater (Majlander et al., 2021), this may reflect the influence of effluents from hospitals connected to NO1 and GR1.

**Figure 7:**
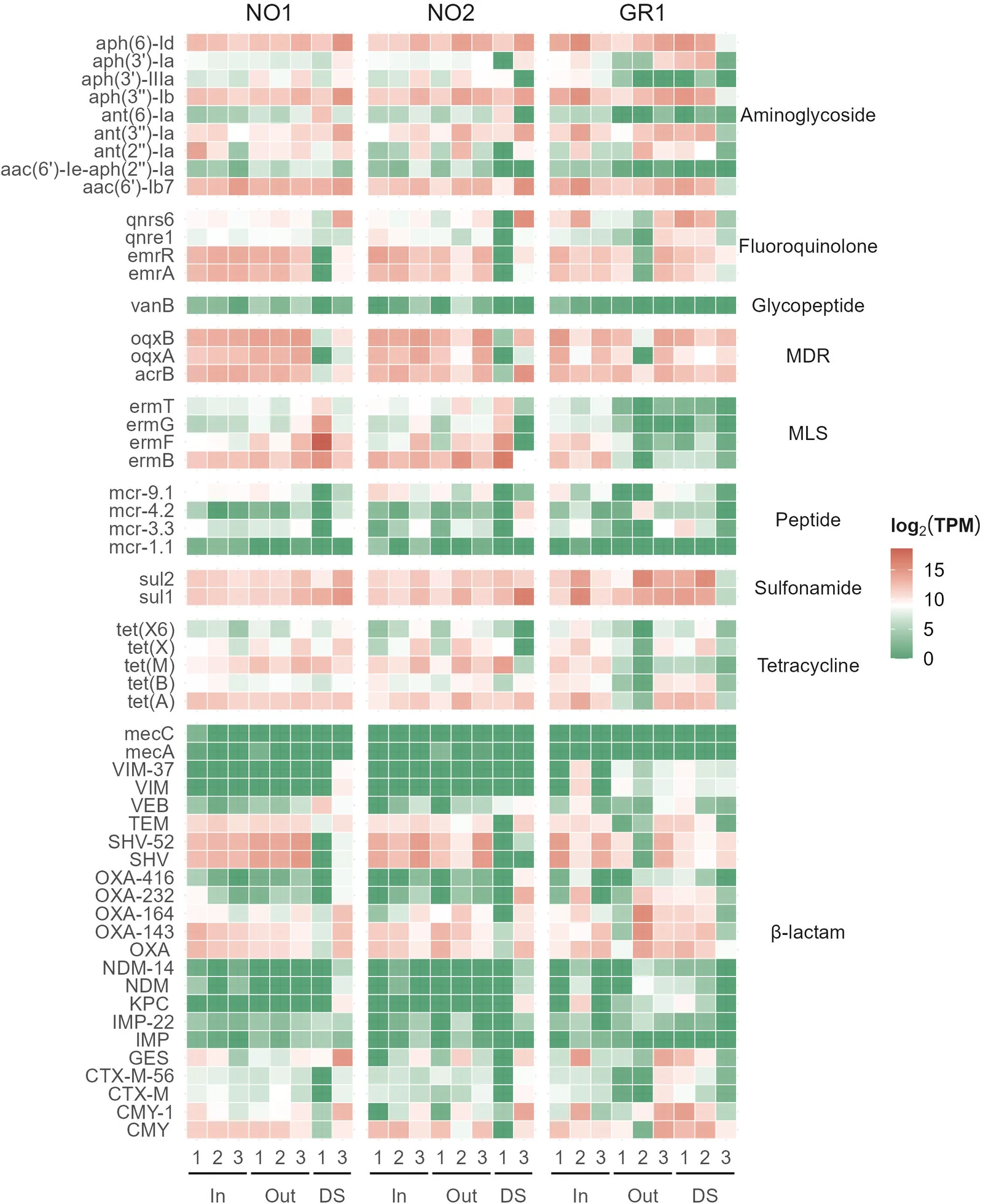
Relative abundance of selected ARGs in inlet (n=9), outlet (n=9), and digested sludge (n=7) samples from NO1, NO2, and GR1. The β-lactam group includes cephalosporin and carbapenem resistance genes. No results were obtained for DS samples from NO1 and NO2 at time point 2.

The relative abundances of selected resistance genes, based on clinical importance and mobility (Zhang et al., 2022), as well as their relative abundance, were investigated more closely. Except for digested sludge at GR1, time point 3, the sulfonamide genes *sul1* and *sul2* (mean relative abundances of 1.2 and 0.8%) were most abundant in all samples (**Figure 7**), consistent with previous findings (Wang et al., 2020). Both *sul1* and *sul2* are highly mobile (Zhang et al., 2022), and co-localization with other ARGs is frequently detected (Shintani et al., 2023; Li et al., 2024). Among the β-lactamase genes, *bla*_OXA_ (7.3 ± 10.7%), *bla*_TEM_ (0.1 ± 0.1%), *bla*_SHV_ (1.6 ± 1.8%), and *bla*_CMY_ (0.7 ± 0.8%) were more abundant than *bla*_VEB_, *bla*_NDM_, *bla*_KPC_, and *bla*_IMP_, which all accounted for < 0.05% of the total resistomes. Despite being less abundant, the presence of genes conferring carbapenem resistance remains a concern, especially given the increasing incidence of bloodstream infections with carbapenem resistant *K. pneumoniae* (ECDC, 2024b). Of the 47 distinct *bla*_OXA_ genes detected, several belong to families known to exhibit carbapenemase activity. The genes *bla*_OXA-232_ and *bla*_OXA-416_ (belonging to the *bla*_OXA-48-like_ family), frequently associated with *Enterobacterales*, and *bla*_OXA-164_, (belonging to the *bla*_OXA-58-like_ family), associated with *Acinetobacter baumanii* (Hamidian and Nigro, 2019; Boyd et al., 2022), were detected at all three WWTPs. This is consistent with a previous study where both *bla*_OXA-48_ and *bla*_OXA-58_ were detected at several WWTPs across Europe (Cacace et al., 2019). These genes were present in outlet samples from all three WWTPs. However, quantitative measurements are needed to evaluate removal rates and the quantities discharged to the environment.

The relative abundance of the selected tetracycline and aminoglycoside resistance genes varied between genes and across samples (**Figure 7**). The widespread resistance genes *tet*(A) and *tet*(M) were detected at high relative abundance in most samples. While *tet*(X*)* (0.1 ± 0.1%) and *tet*(X6) (0.02 ± 0.03%) were less abundant, the presence of these genes in wastewater environments has been reported as increasing (Dai et al., 2025), which is of particular concern as they confer resistance to the last-resort antibiotic tigecycline. Whereas the relative abundance of most of the selected genes remained similar across sample types, the *erm* (erythromycin ribosomal methylase) family, conferring macrolide resistance, typically decreased in relative abundance from inlet to outlet and digested sludge at GR1, in line with previously reported observations of decrease in absolute abundance of *ermB* as well as the abundance of *ermB* relative to the 16S rRNA gene copy number (Rodriguez-Mozaz et al., 2015). A similar trend was observed for *aph(3’)-IIIa* and *aac(6’)-Ie-aph(2’’)-Ia*, which are associated with kanamycin and gentamicin resistance in *Enterococcus* spp (Ben Said et al., 2015). This coincided with a reduction in the mean relative abundance of *Enterococcus* from 0.2 to 0.05%, suggesting that the observed changes could be associated with changes in the bacterial community composition, including a decrease in the relative abundance of potential ARG hosts. This pattern was, however, not observed for most of the selected ARGs.

Resistance to the last-resort antibiotic colistin is of critical importance. The relative abundance of these genes was low compared to other resistance genes. The *mcr-1* (*mcr-1.1*) gene was most frequently detected in inlet samples, but it was also detected in the outlet of NO1 and NO2 at time point 3, in agreement with a previous Norwegian WWTP study (Schwermer and Uhl, 2018). Vancomycin resistance genes were also relatively rare. The clinically important gene *vanA* was not detected in any samples, but the presence of *vanH* and *vanR*, which are located in the same gene cluster, indicates an occasional presence of the *vanA* gene cluster at all three WWTPs. Additionally, *vanB* (< 0.001%) was detected in low abundances at all three WWTPs, but was not detected in sludge and outlet samples at GR1, consistent with previous observations of sporadic *vanB* presence in the outlet of few WWTPs (Pärnänen et al., 2019). Contrarily, the multidrug resistance genes *oqxA* (0.5 ± 0.5%) and *oqxB* (1.1 ± 0.8%) were abundant in most samples, suggesting that they are more widespread in wastewater communities. Their high relative abundance in most outlet and some digested sludge samples shows that multidrug resistance genes are being discharged into the environment via treated wastewater and digested sludge.

### 3.6 Bacterial loads in wastewater discharges and digested sludge depend on wastewater treatment

**Figure 8:**
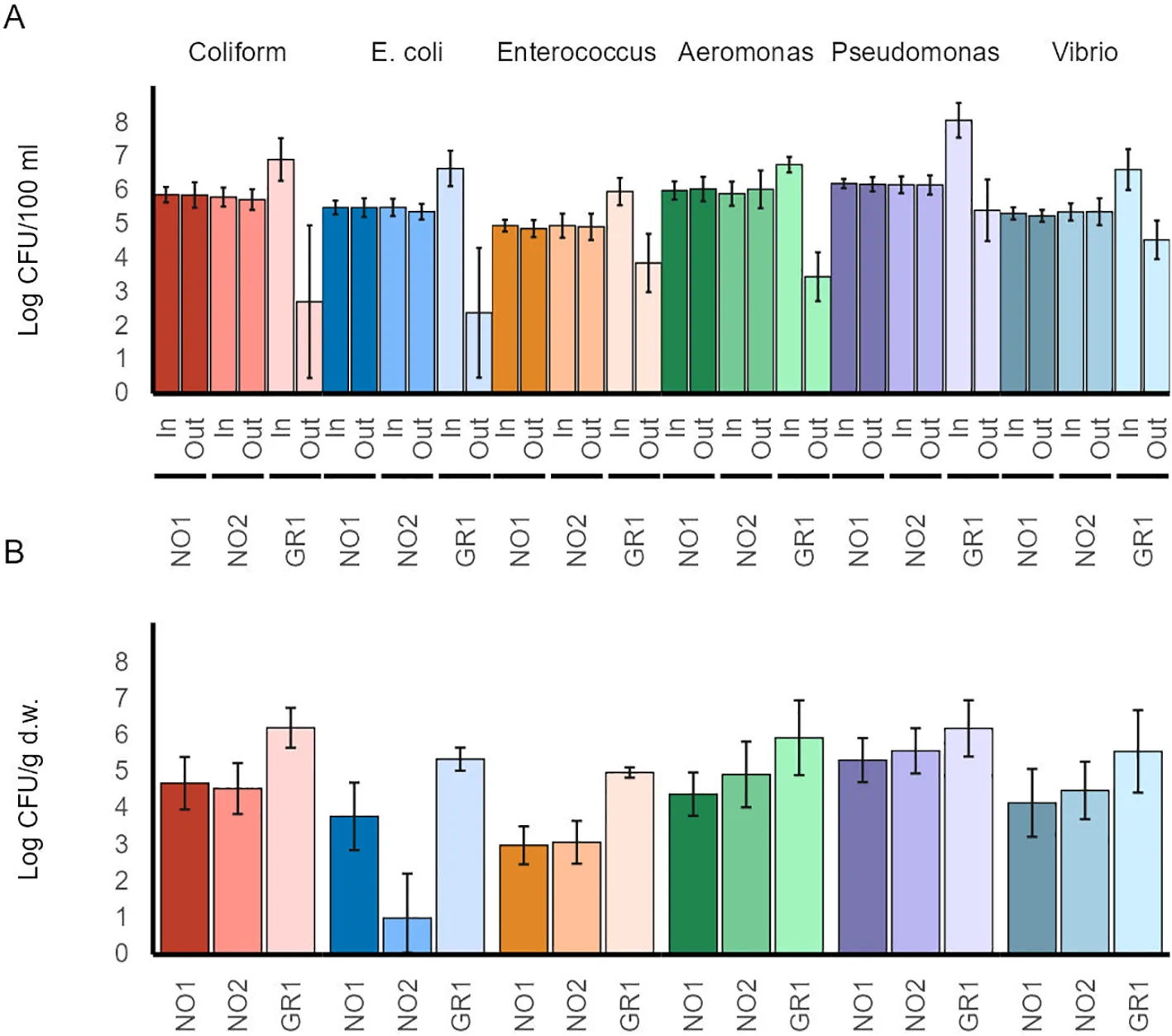
Counts of presumptive fecal (coliform, *E. coli*, *Enterococcus*) and environmental (*Aeromonas, Pseudomonas, Vibrio*) indicator bacteria in **(A)** wastewater inlets (n = 26) and outlets (n = 27), and **(B)** digested sludge (n = 27). Vertical error bars represent ± SD. When counts were below the detection limit, the log CFU was set to 0.

Wastewater (inlet, outlet) and digested sludge samples were plated on selective agars for the enumeration of presumptive fecal (coliform*, E. coli, Enterococcus*) and environmental (*Aeromonas, Pseudomonas, Vibrio*) indicator bacteria. Mean inlet concentrations were similar at NO1 and NO2 for all indicator bacteria, ranging from 4.9 ± 0.4 log CFU/100 mL for *Enterococcus* at NO2 to 6.2 ± 0.1 log CFU/100 mL for *Pseudomonas* at NO1 (**Figure 8**). The reduction in fecal indicators from inlet to outlet was less than 0.2 log CFU/100 mL at both treatment plants, which in practice is negligible. Changes in the concentrations of environmental indicator bacteria were equally small. While mean inlet concentrations at GR1 were significantly higher (Dunn’s test, p < 0.01), exceeding those at NO1 and NO2 by an average of 1.2 ± 0.4 log CFU/100 mL, the outlet concentrations were significantly lower (p < 0.01). The mean reduction by treatment at GR1 was 3.1 ± 1.4 log CFU/100 mL, with the largest reduction of 4.3 ± 1.5 log CFU/100 mL observed for presumptive *E. coli*. The differences may largely be explained by the different treatment technologies at NO1 and NO2 compared to GR1.

In digested sludge, mean concentrations of all indicator bacteria, except for presumptive *E. coli* and *Pseudomonas*, were significantly higher at GR1 than NO1 and NO2 (Dunn’s test, p < 0.05; **Figure 8**). While mesophilic digestion, which is applied at GR1, has a limited eliminatory effect on *E. coli*, pasteurization effectively reduces the concentrations of *E. coli* in wastewater sludge (Lang and Smith, 2008). Both NO1 and NO2 apply pasteurization and normally have acceptable numbers of *E. coli* present in digested sludge, with several tons used for agricultural purposes each year. Equally important as the presence of potential pathogens is the removal of ARB. A decrease in ARB (measured as ARG/16S rRNA copies) has been reported in the case of mesophilic digestion alone (Zahedi et al., 2022). However, the same study reported higher rates of removal for certain genes when pasteurization was applied, indicating that pasteurization may also increase the removal rates of ARB in digested sludge.

A good agreement between agar-based identification and identification based on 16S rRNA gene sequencing among fecal indicator bacteria was observed, with an agreement rate exceeding 85% for coliforms, *E. coli*, and *Enterococcus.* In contrast, the agreement rates for the environmental indicator bacteria were variable, ranging from 0 - 85% (**Supplementary Table 11**), underscoring that culture-based quantification should be accompanied with DNA-based analyses, and that more suitable cultivation methods are needed for the isolation of these bacteria. As discussed by Milligan et al. (2023), this could be especially important in the view of environmental AMR surveillance.

### 3.7 Indicator bacteria exhibit reduced susceptibility to several antibiotics

**Figure 9:**
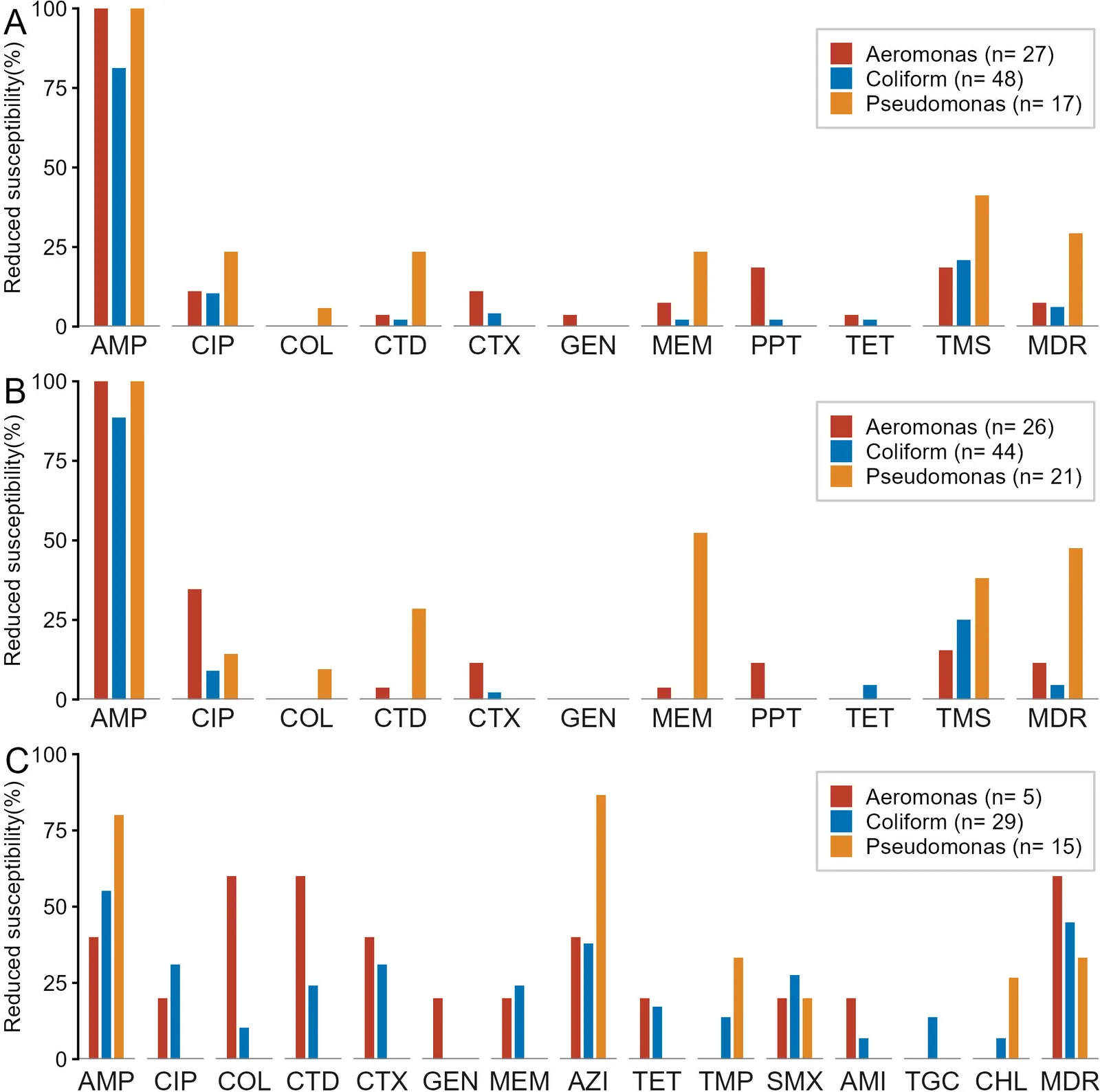
Proportions of isolates showing reduced susceptibility to 10 (NO1 and NO2) or 14 (GR1) antibiotics and proportions of MDR at **(A)** NO1, **(B)** NO2, and **(C)** GR1. Isolates were classified with reduced susceptibility when MIC > ECOFF or, if ECOFF was not available, when the MIC exceeded the S/I clinical breakpoint. AMP, ampicillin; CIP, Ciprofloxacin; COL, Colistin; CTD, Ceftazidime; CTX, Cefotaxime; GEN, Gentamicin; MEM, Meropenem; PPT, Piperacillin/tazobactam; TET, Tetracycline; TMS, Trimethoprim/sulfamethoxazole; MDR, multi-drug resistance; AZI, Azithromycin; TMP, Trimethoprim; SMX, Sulfamethoxazole; AMI, Amikacin; TGC, Tigecycline; CHL, Chloramphenicol.

Selected isolates (n = 279), classified as *E. coli*, coliform, *Enterococcus*, *Aeromonas*, or *Pseudomonas* based on 16S rRNA gene sequencing, were subjected to antibiotic susceptibility testing. It is important to consider that NO1 and NO2 isolates were tested against 3 concentrations for each antibiotic, potentially leading to underestimation of reduced susceptibility rates when the cut-off fell between two of the assay concentrations (**Table 2**, **Supplementary Table 5**). See supplementary **Supplementary Table 4** for individual operational MICs and MICs. Among the tested isolates, reduced susceptibility to all the tested antibiotics was observed (**Figure 9**). Isolates exhibiting reduced susceptibility to at least one antibiotic were detected in all sample types at all three WWTPs. Reduced susceptibility was most frequently observed for ampicillin (86%), followed by azithromycin (53%), the latter being tested only with isolates from GR1. Reduced susceptibility to trimethoprim-sulfamethoxazole at NO1 and NO2 (25%), and sulfamethoxazole at GR1 (25%) was also prevalent, while reduced susceptibility to gentamicin rarely was observed (0.9%), and only in *Aeromonas* isolates. Reduced susceptibility to the 3^rd^ generation cephalosporins cefotaxime and ceftazidime was observed in 11 and 10% of the isolates, respectively. A similar rate of reduced susceptibility (12%) was observed for the carbapenem meropenem. Reduced susceptibility to ≥ 1 antibiotic belonging to ≥ 3 antibiotic drug classes (MDR) was observed in 20% of all isolates and represented by all sample types at all WWTPs.

While there was no significant difference in overall reduced susceptibility rates between WWTPs for most antibiotics, ampicillin, cefotaxime, and MDR differed between the WWTPs (Fisher’s exact test, p < 0.05). The highest rates of reduced susceptibility to ampicillin were observed at NO1 and NO2, while the highest rates of reduced susceptibility to cefotaxime was observed at GR1 (p < 0.05). Furthermore, the rate of MDR was significantly higher at GR1 compared to NO1 and NO2 (p < 0.05). These differences could partially be due to differences in the number of tested isolates of each bacterial group. Coliform isolates from GR1 exhibited reduced susceptibility to most antibiotics tested across WWTPs and at the highest frequencies. Remarkably, only the coliforms from GR1 exhibited reduced susceptibility to colistin (10.3%). The rates of reduced susceptibility to ceftazidime, cefotaxime, and meropenem were 24, 31, and 24%, respectively, while the rates observed at NO1 and NO2 were < 5%. In comparison, resistance rates in *E. coli* isolates from outlet wastewater, when based on ECOFFs, were previously reported to be < 10% for ceftazidime and cefotaxime and < 5% for meropenem (Dioli et al., 2023). Although the resistome of a WWTP is suggested to be stable over long time-scales (Brinch et al., 2020), there is a lack of long-term temporal data on ARB prevalence and a reduction in susceptibility since the previous studies were conducted cannot be excluded. Nevertheless, the higher rates of reduced susceptibility among coliform isolates from GR1 appears to reflect the higher antibiotic consumption rates and antibiotic resistance rates in Greece compared to Norway (ECDC, 2024a, b), rather than the antibiotic residues in wastewater and digested sludge (Section 3.1).

Reduced susceptibility rates among *Aeromonas* isolates were also highest at GR1, while reduced susceptibility rates in *Pseudomonas* isolates varied more across WWTPs depending on the specific antibiotic. The prevalence of reduced susceptibility to colistin and ceftazidime among *Aeromonas* isolates from GR1 was 60%, compared to 0% for colistin and < 5% for ceftazidime among isolates from NO1 and NO2. Furthermore, 60% of *Aeromonas* isolates from GR1 exhibited MDR, compared to 7 and 12% among isolates from NO1 and NO2, respectively. This is slightly lower than previously reported MDR prevalence in treated wastewater and downstream environments (Harnisz and Korzeniewska, 2018). While the number of *Aeromonas* isolates from GR1 (n = 5) was low, it is important to acknowledge their potential as environmental reservoirs of ARGs. *Aeromonas* has an extensive capacity for uptake of genes through HGT and, unlike the fecal coliform bacteria, *Aeromonas* is well adapted to survival in aquatic or soil environments downstream of WWTPs (Zhong et al., 2019; Lamy et al., 2022). In contrast to *Aeromonas*, reduced susceptibility to several antibiotics, including ceftazidime, meropenem, and colistin among *Pseudomonas* isolates, was only observed at NO1 or NO2. Due to insufficient growth at the recommended assessment temperature (35 °C), most of the strains from NO1 and NO2 were assessed at 25 °C. It is known that incubation temperature affects antibiotic susceptibility in *Pseudomonas*, potentially by altering outer membrane permeability (Papapetropoulou et al., 1994; Kitahara et al., 1997). Additionally, the lack of species-specific ECOFFs make interpretation difficult. Within the *Pseudomonas* genus, ECOFFs are mainly given for *P. aeruginosa*, which may lead to the underestimation of resistance in non-*aeruginosa* strains, as wild-type *P. aeruginosa* exhibit elevated MIC for several antibiotics, due to the low outer membrane permeability, efflux pumps, and the activity of antibiotic-degrading enzymes (Pang et al., 2019). Thus, environmental surveillance of ARB depends on an extension of the current ECOFF framework.

**Figure 10:**
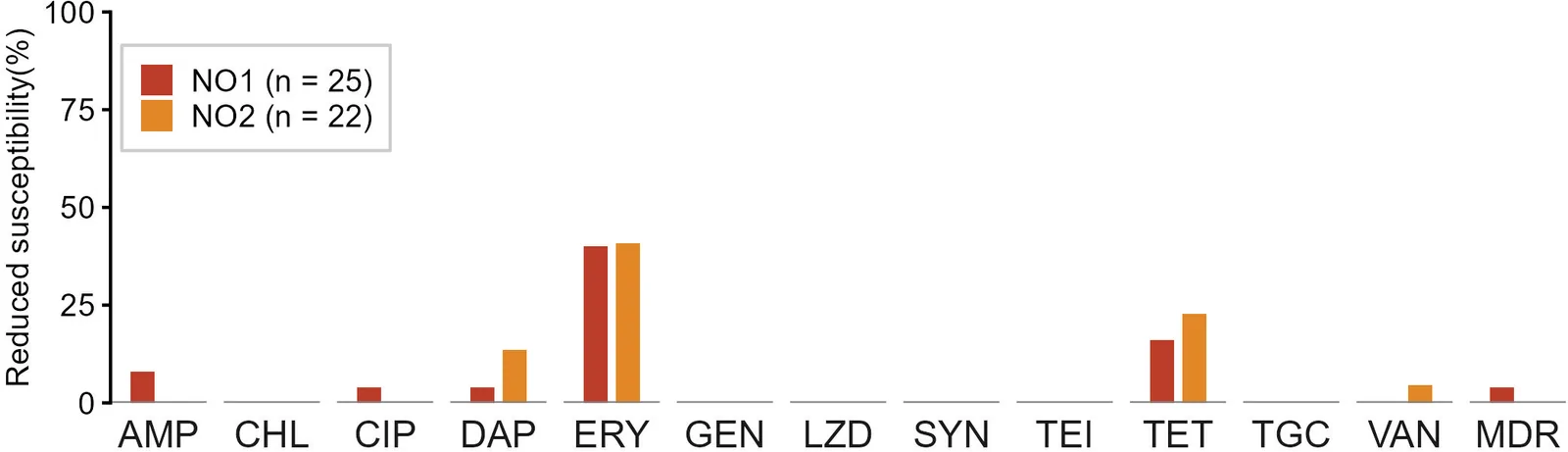
Proportions of isolates identified as *Enterococcus* sp. showing reduced susceptibility to 12 antibiotics and proportions of MDR isolates at NO1 and NO2. Isolates were classified with reduced susceptibility when MIC > ECOFF. AMP, ampicillin; CHL, Chloramphenicol; CIP, Ciprofloxacin; DAP, Daptomycin; ERY, Erythromycin; GEN, Gentamicin; LZD, Linezolid; SYN, Quinupristin/dalfopristin; TEI, Teicoplanin; TET, Tetracycline; TGC, Tigecycline; VAN, Vancomycin; MDR, Multi-drug resistance.

The prevalence of reduced susceptibility among *Enterococcus* from NO1 and NO2 was also assessed (**Figure 10**). The highest rate of reduced susceptibility was observed for the macrolide erythromycin (41%). In line with this, high rates of reduced susceptibility to erythromycin among *Enterococcus faecium* from a Norwegian WWTP has been reported in a previous study (Radisic et al., 2024). They also observed the presence of the macrolide resistance genes *ermB* and *ermT* in several of the strains. Although we did not determine the genetic basis of the observed phenotypes, high relative abundances of *erm* antibiotic resistance genes were observed in wastewater and digested sludge from NO1 and NO2, which could be associated with the observed phenotypes. Reduced susceptibility to daptomycin, erythromycin, and tetracycline was observed at both NO1 and NO2, while reduced susceptibility to ampicillin and ciprofloxacin was only observed at NO1. Additionally, one *Enterococcus gallinarum* isolate from NO2 exhibited reduced susceptibility to vancomycin. *E. gallinarum* is intrinsically resistant to vancomycin due to a chromosomally encoded *vanC* gene cluster (Arias et al., 2000). In the previous study on antibiotic resistance among *E. faecium* from a Norwegian WWTP, resistance to ciprofloxacin and tetracycline was also detected (Radisic et al., 2024). In contrast, they also detected a high prevalence of quinupristin/dalfopristin resistance (68%) and no resistance to daptomycin. The isolates exhibiting reduced susceptibility to daptomycin were all classified as *Enterococcus hirae*, while the previous study only investigated *E. faecium*. Similar to our observations, resistance to daptomycin has been observed to be more common among *E. hirae* than other *Enterococcus* species (Cho et al., 2020). However, it remains unclear whether this reflects intrinsically higher MIC, species-specific resistance mechanisms, or acquired resistance determinants. Low MDR rates of 0 and 4% for NO1 and NO2 were observed, indicating that decreased susceptibility to multiple antibiotics is uncommon among *Enterococcus* isolates at NO1 and NO2.

While the prevalence of reduced susceptibility to certain antibiotics and the rate of MDR among fecal and environmental indicator bacteria were higher at GR1 compared to NO1 and NO2 (Fisher’s exact test, p < 0.05), the bacterial loads in the wastewater effluents of NO1 and NO2 were significantly higher for fecal bacteria and *Aeromonas*. Consequently, the discharge of non-susceptible bacteria could be higher at NO1 and NO2. This is particularly concerning, considering the presence of bacteria exhibiting reduced susceptibility to important antibiotics such as colistin, ceftazidime, cefotaxime, meropenem, and daptomycin at NO1 and NO2.

### 3.8 Implications and limitations

The widespread occurrences of antibiotic residues, PTMs, ARGs, and bacteria exhibiting reduced antibiotic susceptibility across the three WWTPs supports that WWTPs are reservoirs of AMR. Furthermore, the detection of these components in outlet wastewater samples at all three WWTPs demonstrates their dissemination into marine environments, also in the low antibiotic consumption and clinical AMR prevalence areas represented by NO1 and NO2. The high concentrations of antibiotic residues and indicator bacteria in the outlets at NO1 and NO2, resulting from limited reduction by treatment, highlights the inefficiency of primary wastewater treatment at reducing antibiotic residue concentrations and bacterial loads from wastewater, as previously reported (Francy et al., 2012; Guerra et al., 2014). Alongside efforts to decrease antibiotic consumption, improvements in the wastewater treatment targeting degradation and/or removal of antibiotics and bacteria could therefore decrease their discharge to marine environments. While current studies of WWTPs in relation to AMR often focus on wastewater, our findings highlight a need for greater attention to digested sludge as a potential environmental dissemination route for AMR. Although increases in ARG abundance downstream of wastewater effluents and after biosolid application have been observed (Qin et al., 2022; Read et al., 2024), the risk associated with environmental discharge of ARB and ARGs is not fully understood. Thus, improved risk assessment frameworks could aid a better evaluation of the potential human health implications associated with the observed environmental dissemination of ARGs and bacteria exhibiting reduced susceptibility to antibiotics.

Study design limitations of this work should also be considered. The investigation of WWTPs located in areas with contrasting antibiotic consumption and clinical resistance rates enabled the exploration of how this is reflected in wastewater. However, the location of the WWTP and wastewater treatment configuration were inherently confounded, making it challenging to disentangle location and treatment-related effects. This was particularly relevant for the ARG analyses, where the limited number of pooled samples precluded assessment of interaction between location and sample type, and for the antibiotic susceptibility analyses, where prevalence was assessed at the WWTP level rather than separately for each sample type. Hybrid capture-based sequencing provides a sensitive method for ARG detection, especially in wastewater with low ARG abundance (Lindstedt et al., 2025). At the same time, the reliance on pre-defined probes prohibits detection of novel ARGs, and variation in enrichment efficiency may bias gene abundance estimates (Salmon et al., 2012). Additionally, this approach does not consider the genetic context of the ARGs, preventing their association with bacterial hosts or mobile genetic elements. Such information provide insight into clinical relevance and dissemination potential, and is thus important when evaluating ARGs within a risk-assessment framework (Klümper et al., 2025). The lack of quantitative ARG measurements also limited per-gene comparisons across WWTPs and sample types. While shotgun metagenomic sequencing could provide this information, it would likely come at a cost of reduced sensitivity. Finally, although the AST enabled resistance phenotype to be linked to bacterial hosts, genomic characterization of isolates exhibiting reduced susceptibility was not performed. Consequently, the genetic basis of the observed phenotypes, as well as the pathogenic potential could not be determined.

## 4 Conclusion

Through a multifaceted assessment of bacterial communities, selective agents, and AMR indicators, this study investigated the state of AMR at three urban WWTPs in Norway and Greece. Bacterial community composition was influenced by WWTP location, sample type, and sampling time point, emphasizing the importance of spatial and temporal variations in shaping wastewater microbiomes. Furthermore, antibiotic residues and PTMs were detected in wastewater and digested sludge at concentrations exceeding PNECs and MSCs, and significant associations between several antibiotic residues and bacterial community composition were observed. ARGs and bacteria exhibiting decreased antibiotic susceptibility were detected across all WWTPs, and their environmental dissemination through wastewater effluents and digested sludge was demonstrated. This may have implications for human health, although evaluation of the associated risk is hampered by the lack of risk assessment frameworks. Nevertheless, effective wastewater treatment and management alongside responsible antibiotic use could limit the environmental dissemination of AMR. The combined application of a highly sensitive resistome analysis and phenotypic susceptibility profiling provided a robust and complementary framework for AMR assessment in wastewater systems, strengthening these findings. Overall, this study demonstrates the important role of WWTPs in the environmental dissemination of AMR and emphasizes the need for integrated surveillance and mitigation strategies that link human and environmental sectors within a One Health framework.

## Data Availability

The raw sequencing data generated in this study have been deposited in the NCBI Sequence Read Archive (SRA) under the BioProject accession number PRJNA1481110.
