# Supplementary material for: Urban wastewater and sewage sludge as environmental sources of antimicrobial resistance, potential pathogens, and selective agents: insights from three European wastewater treatment plants
Source: Front Cell Infect Microbiol. 2026 Sep 10;16:1916091. doi: 10.3389/fcimb.2026.1916091 (PMC13600862; doi:10.3389/fcimb.2026.1916091)
Supplement: Supplementary file 1 [file Table1.docx]

# Supplementary Figures

Figure S1: Rarefaction curves for inlet, outlet, and digested sludge samples from the three WWTPs. Curves were constructed with the rarecurve() function in R with a step size of 50 and a subsample size equal to the reads in the sample with least amount of reads. The endpoint of each curve represents that samples sequencing depth after quailty and chimera filtering. Sample refers to number of reads and Species refer to number of OTUs.

Figure S2: Venn diagrams for the different sampletypes (inlet, outledoutlet and digested sludge) at the three WWTPs. Numbers represent number of ARGs and percentage represents the percentage of all ARGs detected at the specific WWTP.
