# Supplementary material for: Urban wastewater and sewage sludge as environmental sources of antimicrobial resistance, potential pathogens, and selective agents: insights from three European wastewater treatment plants
Source: Front Cell Infect Microbiol. 2026 Sep 10;16:1916091. doi: 10.3389/fcimb.2026.1916091 (PMC13600862; doi:10.3389/fcimb.2026.1916091)
Supplement: Supplementary file 2 [file Table2.docx]

# Supplementary Methods

**MS settings:**

The instrument was operated in positive bbCID mode equipped with an ESI source with an end plate offset of 500 V and a capillary voltage of 4500 V. The dry gas flow and temperature were set to 10.0 L/min and 220 °C, respectively, and the nebulizer pressure was 2.2 bar. Funnel 1 RF and Funnel 2 RF were both set to 200 Vpp, the hexapole RF was 50 Vpp, and the quadrupole ion energy was 5.0 eV. The collision cell energy was set to 5.0 eV, with a pre-pulse storage time of 5.0 µs. Spectra were acquired in MS mode over an *m/z* range of 75–1000 at a rate of 2.0 Hz, using a rolling average of 2 spectra. The low-mass cutoff was set to *m/z* 50, and the isCID energy was 0 eV. The fragmentation energies for the bbCID was 6 eV for full scan and 30 eV for the bbCID spectra.

**HPLC method**

HPLC separation was achieved by a Waters HSS T3 C18, 2.1x100 mm operated at 40C at a flow rate of 0.40 mL min⁻¹ using solvent A (water containing 2 mM ammonium formate) and solvent B (acetonitrile containing 2 mM ammonium formate). The gradient was programmed as follows: 0–4 min, 0.5% B; 4–10 min, linear increase to 40% B; 10–18 min, linear increase to 100% B; 18–21 min, held at 100% B; 21–21.5 min, returned to 0.5% B and re-equilibrated. The total run time was 22 min with a post-run equilibration time of 3 min. Injection volume was 10 µL for all samples, standards and system blanks.

**Quantification**

Quantification was done in TASQ 2026.1.4 (Bruker Daltonics) from both Full Scan and bbCID data  with mandatory and quantifier ions enabled. Setting was equal for both fullscan and bbCID data. Peak detection required a minimum area threshold of 500 and a height threshold of 200, with a sensitivity setting of 99%. Minimum peak valley was set to 4.0%. Extracted ion chromatograms (EICs) were generated using a mass window for narrow windows of ± 3.0 ppm and wider windows ± 7.0 ppm. Retention time for narrow window was ± 0.25 min and 0.4 min for wide window.  All hits fulfilling narrow settings for retention time, mass accuracy, isotope pattern (mSigma < 40) and found all diagnostic peaks were automatically processed while any deviations from this required a manual approval or disapproval of the hit prior to quantification. All calibration curves were based on chromatographic peak and concentrations run from LLOQ to 2270 nmol/L, R^2^ for the quadratic calibration curves with 1/x weighting were 0.999 or better.

**Figure MS1:**  Chromatographic separation of standards, all scaled to 100% to show all antibiotics in the same chromatogram.

**Table MS1:** retention time and LLOQ of the investigated antibiotics.

| **Analyte** | **Expected RT (min)** | **LOQ (nmol/L)** |
| --- | --- | --- |
| Amoxicillin | 6.4 | 227 |
| Vancomycin | 7.1 | 227 |
| Meropenem | 7.3 | 2.3 |
| Sulfapyridine | 7.7 | 2.3 |
| Ampicillin | 7.8 | 22.7 |
| Trimethoprim | 8 | 0.23 |
| Cefotaxime | 8.1 | 22.7 |
| Ciprofloxacin | 8.2 | 2.3 |
| Levofloxacin | 8.3 | 2.3 |
| Mecillinam | 8.4 | 0.23 |
| Cefazolin | 8.4 | 22.7 |
| Tetracycline | 8.5 | 2.3 |
| Sulfamethoxazole | 9.4 | 0.23 |
| Azithromycin | 9.6 | 2.3 |
| Clindamycin | 9.6 | 2.3 |
| Piperacillin | 10.5 | 22.7 |
| Penicillin G | 10.7 | 22.7 |
| Erythromycin | 10.9 | 22.7 |
| Penicillin V | 11.2 | 22.7 |
| Cloxacillin | 12.1 | 22.7 |
| Dicloxacillin | 12.7 | 22.7 |
